# Computational approaches to deep learning–driven EEG analysis for neurological disease diagnosis: a systematic review

**DOI:** 10.3389/fninf.2026.1778980

**Published:** 2026-10-13

**Authors:** Thazneem H F, Jisu Elsa Jacob

**Affiliations:** 1 Sree Chitra Thirunal College of Engineering, Pappanamcode, Thiruvananthapuram, Kerala, India; 2 Research Scholar, APJ Abdul Kalam Technological University, Kerala, India

**Keywords:** automated diagnosis, deep learning-artificial intelligence, EEG, EEG analysis methods, machine learning-based classification

## Abstract

Electroencephalography (EEG) is a widely used non-invasive neurophysiological technique for analyzing brain dynamics and diagnosing neurological diseases. However, the non-stationary nature, low signal-to-noise ratio, and inter-subject variability of EEG signals cause significant challenges to traditional analytical approaches. In recent years, deep learning has emerged as a powerful computational tool for EEG analysis, which enables automated feature learning and improved diagnostic accuracy with minimal human-driven feature extraction. This review systematically explains computational approaches in deep learning-driven EEG analysis for the diagnosis of neurological diseases. It includes recent advances in deep learning architectures, such as convolutional neural networks (CNN), recurrent neural networks (RNN), hybrid configurations, Generative Adversarial Networks (GAN), Large Language Models (LLM), autoencoders, and transformer-based models, with a focus on their capacity to model spatial, temporal, and spatiotemporal dynamics in EEG signals. This is applicable in diagnosing major neurological disorders such as Epilepsy, Alzheimer’s disease (AD), Encephalopathy, Parkinson’s disease (PD), Mild Cognitive Impairment (MCI), sleep disorders, and developmental brain disorders. Furthermore, we discuss essential preprocessing strategies and denoising techniques, showing how removing biological artifacts prevents diagnostic models from confusing noise with actual signs of disease. Key challenges such as limited data availability, lack of model interpretability, inter-subject variability, and difficulty in clinical translation are identified and critically discussed. Finally, key emerging trends and future research directions are identified to support the development of clinical applications of EEG-based disease diagnostic systems.

## 1 Introduction

Electroencephalography (EEG) is a non-invasive neurophysiological (Chaudary et al., 2025) technique recording the brain’s electrical potentials (Sharma and Meena, 2024) via scalp electrodes (Figure 1A; Everest et al., 2025; Xiong et al., 2025; Zhang et al., 2025, p. 5), enabling real-time monitoring (Cursi and Filippi, 2025) of neuronal dynamics with high temporal precision. It widely supports diagnosing and tracking neurological and psychiatric conditions, including epilepsy (Myers et al., 2025; Weber, 2025), sleep abnormalities (Proserpio et al., 2025), depression, Parkinson’s Disease (PD) (Lee et al., 2025), Mild Cognitive Impairment (MCI), and Alzheimer’s Disease (AD) (Paitel et al., 2025).

**FIGURE 1:**
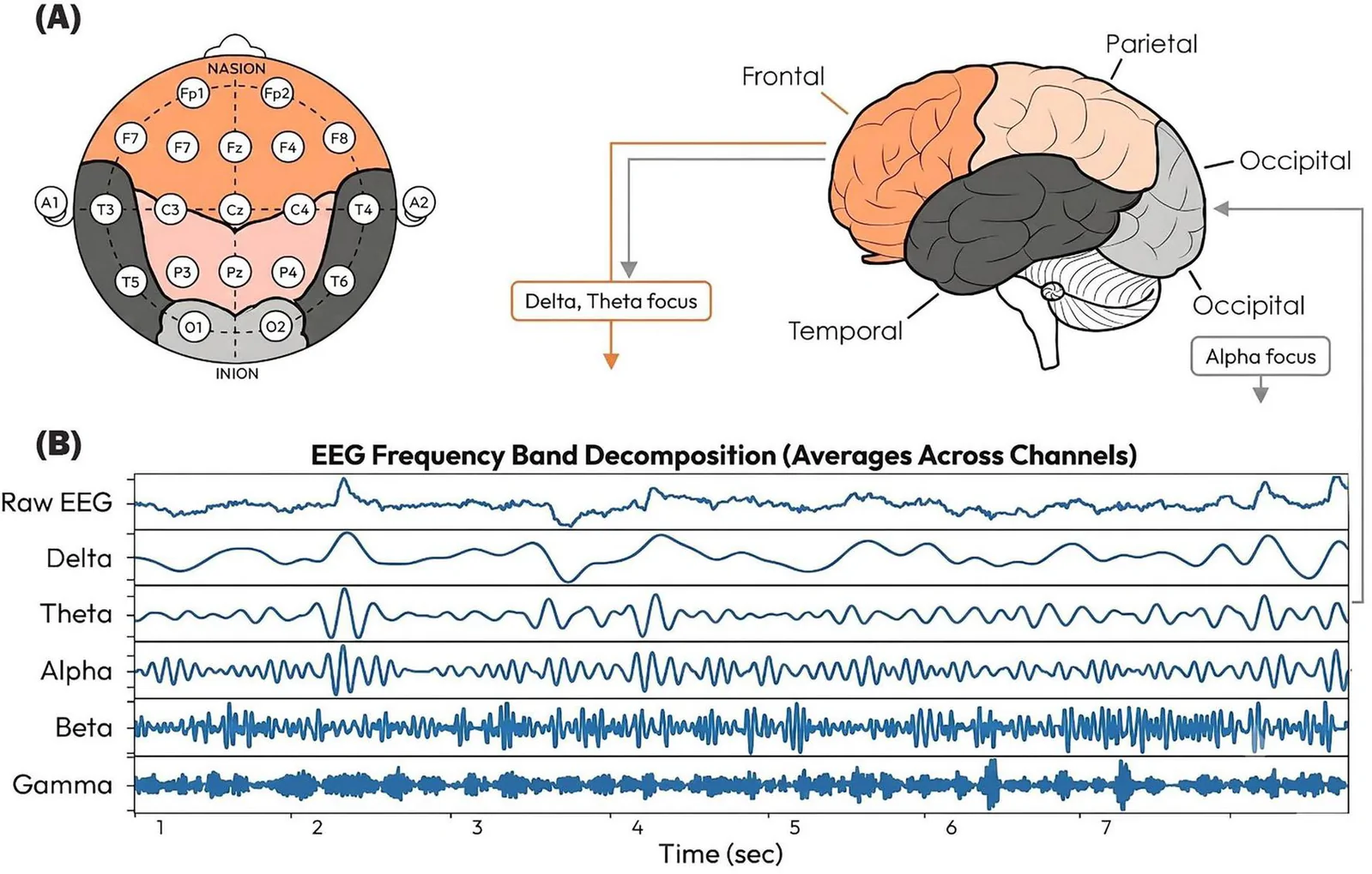
Structural and spectral characteristics of clinical EEG acquisition. **(A)** The electrode layout of the 10-20 international system (left) and corresponding cortical brain regions (right) (Homan et al., 1987). **(B)** EEG recording of a healthy subject over 200 seconds, decomposed into five major frequency bands representing distinct cerebral oscillations (Robinson, 1999).

EEG signals comprise distinct oscillatory rhythms categorized into canonical frequency bands: delta (0.5–4 Hz), theta (4–8 Hz), alpha (8–13 Hz), beta (13–30 Hz), and gamma ( > 30 Hz) (Maaz et al., 2025; Figure 1B and Table 1). Beyond serving as physiological markers, these bands function as critical spatiotemporal features for machine learning pipelines. For instance, localized shifts in theta/alpha ratios (Schmidt et al., 2013; Baik et al., 2022) or degraded gamma bands (Ahn et al., 2013) provide key mathematical indicators used by deep learning networks to classify cognitive decline or detect localized ischemia.

**TABLE 1:** The five bands of EEG signals (Herrmann et al., 1979; Schomer and Lopes da Silva, 2017).

Wave type	Frequency band (Hz)	Typical amplitude (μ V)	Associated brain states
Delta (δ)	0.5–4	20–200	Deep sleep, unconsciousness, restorative processes, dreaming, brain healing, coma states
Theta (θ)	4–8	20–100	Creative thinking, emotional stress, drowsiness, meditation, memory encoding, early stages of sleep
Alpha (α)	8–12	20–60	Relaxation, calm alertness, eyes-closed resting, meditation, wakeful rest, reduced sensory input
Beta (β)	12–30	5–20	Active concentration, alertness, anxiety, problem solving, motor activity, decision making
Gamma (γ)	>30 (typically 30–100)	<10	High-level cognition, learning, perception binding, focused attention, sensory integration, consciousness

However, because scalp EEG operates in the microvolt range, its non-stationary nature leaves it highly vulnerable to contamination from physiological artifacts, including Electrooculography (EOG), Electromyography (EMG), and Electrocardiography (ECG) (Zhou and Gotman, 2004; Chaudhary, 2025) and non-physiological line noise. Traditional linear filters often fail because noise removal can inadvertently suppress pathognomonic neural features.

To preserve signal integrity for AI models, modern neuroinformatics pipelines deploy hybrid signal-processing strategies. Automated Independent Component Analysis (Zhou and Gotman, 2004) coupled with Wavelet Transforms (Wavelet-Independent Component Analysis (ICA) (Sai et al., 2017) isolates high-amplitude ocular (EOG) (Chaudhary, 2025) blinks without removing underlying frontal delta rhythms. Similarly, empirical time-frequency decompositions such as Variational Mode Decomposition (VMD) (Dragomiretskiy and Zosso, 2013) or Fourier–Bessel Series Expansion Empirical Wavelet Transform (FBSE–EWT) (Bhattacharyya et al., 2018) isolate complex, broadband muscle (EMG) artifacts (Zhou and Gotman, 2004). These advanced spatial-filtering techniques prevent machine learning models from learning artifact patterns, ensuring deep networks extract legitimate, clinically relevant neural correlates.

Deep learning (DL) has transformed EEG denoising by learning nonlinear, data-driven mappings from noisy signals to clean targets without reference channels. Early DL methods applied CNNs (Chaudary et al., 2025) or RNNs (Sreedhar et al., 2025) to identify artifact components within ICA frameworks. Conversely, recent end-to-end architectures including autoencoders (AEs) (Abdi-Sargezeh et al., 2025), Generative Adversarial Networks (GANs) (Tibermacine et al., 2025), transformers (Ghous et al., 2025) and hybrid models (Li D. et al., 2024) achieve superior performance. More recently, Large Language Models (LLMs) (Chandrasekharan and Jacob, 2025) and generative frameworks are being explored in EEG neuroinformatics (Kilicarslan et al., 2016). However, their clinical utility requires careful delineation: while automated clinical report generation from pre-extracted features represents a demonstrated finding, cross-modal feature translation and adaptive representation learning remain emerging domains, with direct autonomous diagnosis and clinical decision support remaining prospective future perspectives requiring further empirical support from EEG-specific studies.

### 1.1 Background and significance of EEG in healthcare

Neurological disorders present an escalating global health crisis, currently affecting nearly one billion individuals worldwide (Tatum et al., 2018). Among these, epilepsy accounts for 50 million cases, while neurodegenerative conditions like dementia and Alzheimer’s disease affect over 24 million individuals, standing as the top global causes of mortality (Tatum et al., 2018). As global populations age, the socioeconomic burden (GBD 2019 Mental Disorders Collaborators, 2022) of these conditions is projected to rise sharply across both developed and developing economies, requiring a critical shift toward scalable, early, and automated diagnostic methodologies (GBD 2019 Mental Disorders Collaborators, 2022; Steinmetz et al., 2024).

To track this progression, we need objective, fast-acting biomarkers that can monitor brain degeneration and altered network activity. Electroencephalography (EEG) is ideal for this because it is non-invasive, cost-effective, and captures brain activity in real time. However, manually analyzing complex, ever-changing EEG data across large patient groups is too slow for everyday clinical use. Therefore, automating EEG analysis with advanced machine learning and AI pipelines is essential (Steinmetz et al., 2024; Jonna and Natarajan, 2025). Developing automated diagnostic systems for diseases like Alzheimer’s, Parkinson’s, MCI, and epilepsy is the only way to provide fast, personalized care to everyone who needs it.

### 1.2 Need and challenges for AI in EEG analysis

Evaluating EEG brainwaves traditionally relies on clinicians looking at the data by hand. This process is slow, subjective, and can vary from one expert to another. Because checking hours of complex brain data by hand is impossible at scale, we urgently need fast, automated software (Mao et al., 2020). Automated systems take the burden off medical staff by scanning data to flag irregularities or draft diagnostic summaries for clinicians to double-check. This speeds up treatment and improves patient outcomes. Machine learning and deep learning pipelines (Jonna and Natarajan, 2025) provide a reliable way to automate this process by finding key brain patterns directly within the data. When trained well, these models can match the accuracy of expert medical practitioners while delivering completely consistent results (Alharthi et al., 2025).

However, several major hurdles stop these AI tools from being used in hospitals right now. Because every person’s brain waves are slightly different, models struggle to work accurately when tested on new patients (Koren et al., 2018). Large, properly labeled medical datasets are also very hard to find. Additionally, the complex “black box” nature of deep learning hurts clinical trust, as medical practitioners cannot see exactly how the AI made its decision (Acharya et al., 2013). Moving these tools into actual medical practice requires strict clinical testing, official regulatory approvals, standardizing hardware, and building simple, user-friendly software for hospital staff. Solving these issues is essential to creating accurate, clear, and truly helpful AI tools for personalized patient care.

### 1.3 Objectives and scope of the review

This systematic review provides a computationally driven evaluation of advanced EEG signal processing, focusing on the paradigm shift from traditional analytical frameworks to modern machine learning (ML) and deep learning (DL) architectures in healthcare (Kumar et al., 2022). Aligned with the core methodologies of neuroinformatics, the scope systematically covers the entire processing pipeline, from preprocessing and artifact suppression to feature extraction, selection, and dimensionality reduction. We evaluate the capacity of modern end-to-end networks, including CNNs, RNNs, hybrid topologies, Autoencoders, GANs, Transformers, and emerging LLM-inspired foundational frameworks, to effectively model the complex spatiotemporal dynamics of non-stationary neural time-series data.

Clinically, this work targets the translation of automated computational models for predictive diagnosis across a spectrum of major neurological and neuropsychiatric conditions. Specifically, we highlight deep learning applications in epilepsy, sleep abnormalities, psychiatric conditions, and neurodegenerative pathologies such as Parkinson’s disease (PD) (Lee et al., 2025), Mild Cognitive Impairment (MCI) (Rossini et al., 2022), and Alzheimer’s disease (AD) (Sreedhar et al., 2025). Finally, the review addresses real-world bottlenecks such as data scarcity, inter-subject variability, and model interpretability while outlining future trends in real-time execution and Explainable AI (XAI) to bridge the gap between biophysical signal analysis and clinically integrated precision medicine. While deep learning (DL) architectures have demonstrated remarkable performance on large-scale datasets, they do not universally supplant conventional machine learning (ML) frameworks. Traditional pipelines utilizing feature engineering (e.g., wavelet transforms, spectral entropy, and Hjorth parameters) paired with classical classifiers like Support Vector Machines (SVM) or Random Forests often achieve comparable accuracy (typically 85–91%) on small-sample cohorts with significantly lower computational overhead and superior interpretability (Alqudah and Moussavi, 2025). Conversely, end-to-end DL models, particularly Convolutional Neural Networks (CNNs), excel when scaling to high-density, multi-channel repositories like the TUH EEG database, achieving accuracies upwards of 92–96% at the cost of higher parameter complexity and reduced transparency (Zhu, 2025; Colafiglio, 2026). Thus, the choice between conventional ML and DL represents a trade-off between sample efficiency, computational feasibility, and raw diagnostic capacity rather than an absolute superiority of one approach over the other.

### 1.4 Literature selection strategy and inclusion criteria

To ensure a comprehensive and reproducible selection process, a systematic literature search was executed across PubMed, IEEE Xplore, and Google Scholar for studies published up to December 2025. To address the full clinical scope of this review, a search query was deployed: (“EEG” OR “Electroencephalography”) AND (“Deep Learning” OR “Transformers” OR “Neural Networks”) AND (“Denoising” OR “Feature Extraction”) AND (“Neurological Disorders” OR “Epilepsy” OR “Alzheimer’s” OR “Dementia” OR “Parkinson’s” OR “Encephalopathy” OR “Sleep Disorders” OR “Psychiatric Conditions”).

The multi-stage screening process was performed over a designated search timeline from October 10, 2025, to December 15, 2025. The initial database search yielded 212 records from PubMed, 115 records from IEEE Xplore, and 950 relevance-filtered, peer-reviewed indexings from Google Scholar, totaling 1,277 raw citations (Figure 2). Following the removal of duplicate entries using reference management software (Zotero) (*n* = 312), 965 unique records were screened based on titles and abstracts. This phase led to the exclusion of 720 records that did not focus on computational deep learning architectures or clinical disease diagnosis. Of the remaining 245 full-text articles assessed for detailed eligibility, 228 were excluded. Specifically, 132 papers were excluded due to a non-diagnostic focus, such as standalone Brain-Computer Interfaces (BCIs), motor-imagery control, or affective computing. An additional 96 records were excluded because they constituted general background literature or lacked explicit performance metrics. Ultimately, 17 milestone deep learning papers were preserved for systematic comparative synthesis and state-of-the-art diagnostic benchmarking in Table 3.

**FIGURE 2:**
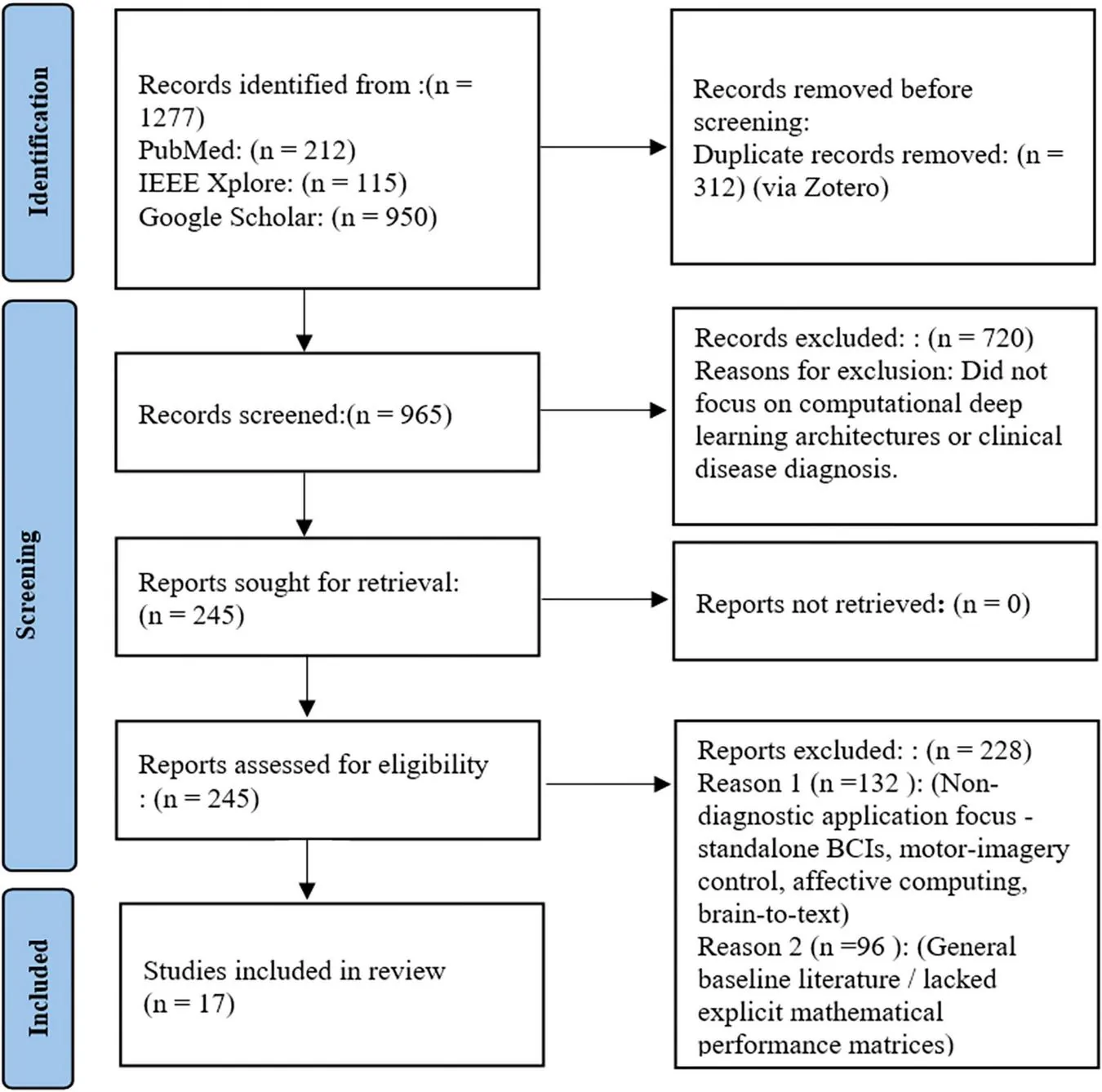
PRISMA flow diagram illustrating the systematic literature selection process.

## 2 Advanced EEG signal processing

EEG signal processing constitutes a multidimensional pipeline designed to transform raw, noisy scalp recordings into high-fidelity representations of neural activity. EEG signals are inherently low-amplitude, non-linear, and highly non-stationary (Paitel et al., 2025), necessitated by the complex, overlapping nature of cortical sources and physiological contamination. To extract meaningful insights for microstate analysis (Khanna et al., 2015), pathognomonic insights for automated clinical diagnostics and machine-learning-based disease classification (Subasi and Gursoy, 2010), modern pipelines must rigorously integrate signal acquisition, artifact suppression, feature engineering, and dimensionality reduction (Subasi and Gursoy, 2010). It ensures that automated diagnostic tools focus on real brain signals rather than misleading noise.

### 2.1 EEG preprocessing and removal of biological artifacts

The analytical pipeline commences with signal acquisition (see Figure 3; Sharma and Meena, 2024), where cortical electrical activity is captured using scalp-based electrodes, typically configured via the international 10–20 or 10–10 system (Figure 1A; Homan et al., 1987). The efficacy of subsequent analysis is fundamentally constrained by the quality of this stage, which is influenced by electrode skin impedance (Li et al., 2017), sampling resolution, and environmental noise (Papatzikis, 2025). During this stage, the raw signal contains both useful neural information and numerous unwanted physiological and non-physiological artifacts, which necessitate further processing.

**FIGURE 3:**
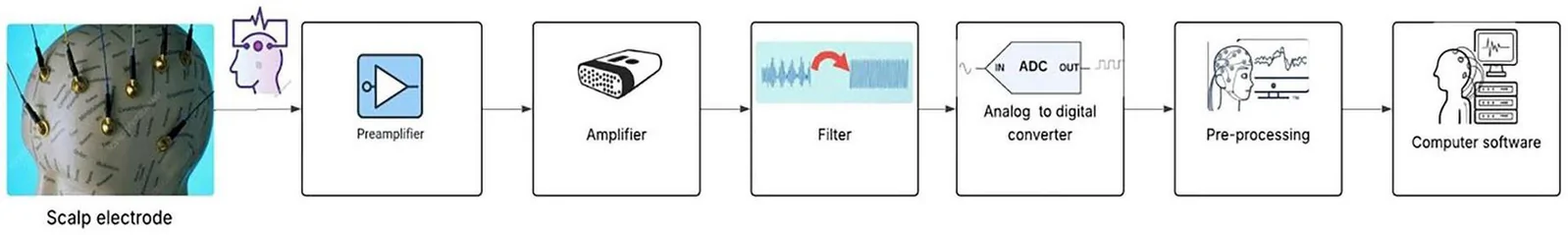
The step-by-step pipeline of the EEG signal acquisition system.

Preprocessing aims to convert raw EEG data into a cleaner and more stable representation that can be reliably analyzed (Sanei and Chambers, 2013). This step typically involves several processes, including band-pass filtering (Lemm et al., 2005) to remove slow drifts and high-frequency noise (Cho et al., 2011), along with re-referencing and resampling when necessary to achieve uniformity.

A major aspect of preprocessing is artifact removal (Urigüen and Garcia-Zapirain, 2015), as EEG signals are easily affected by factors such as ocular movements, blinking, muscle activity, cardiac signals, and electrical interference. Techniques like regression, independent component analysis (ICA) (Jonna and Natarajan, 2025), wavelet thresholding (Krishnaveni et al., 2006), and adaptive filtering (Kher and Gandhi, 2016) are often employed to isolate and suppress these non-stationary biological artifacts (Varanini et al., 1993). Effective preprocessing enhances the signal-to-noise ratio, thereby improving feature extraction and downstream learning algorithms.

A primary challenge in clinical neuroinformatics is that raw scalp recordings contain a mixture of microvolt-level neural oscillations and high-amplitude physiological artifacts (Mannan et al., 2018). Because the skull heavily weakens the brain’s electrical signals, non-brain activities like blinking or muscle movement easily cover up the important patterns medical practitioners look for. Due to the highly non-stationary nature of these signal mixtures, simple linear filtering is insufficient, necessitating specialized artifact gating mechanisms to handle the two most prominent biological confounders.

- Electrooculographic (EOG) Artifacts (Schlögl et al., 2007): Triggered by eye blinks and lateral or vertical ocular movements, EOG artifacts generate high-amplitude voltage shifts up to 100 μV (Chen et al., 2019). These present as low-frequency phenomena (<4 Hz) concentrated heavily across frontal and frontopolar electrode tracks (Fp1, Fp2)
- Electromyographic (EMG) Artifacts (Chen et al., 2019): Caused by cranial muscle activity, jaw clenching, or neck tension, these present as high-frequency noise elements ( > 20 Hz) stretching across a broad spatial distribution, heavily affecting temporal and occipital electrodes.

If left unaddressed, low-frequency eye movements (EOG) can look exactly like the slow brainwaves (delta rhythms) caused by brain disease or physical injuries. On the other hand, muscle tension (EMG) can hide the loss of fast brainwaves (gamma waves) that signal early dementia. When these artifacts are not cleaned up, they confuse automated software and trigger false alarms.

To extract underlying brain dynamics without discarding compromised data epochs, modern preprocessing pipelines deploy blind source separation (BSS) (Urigüen and Garcia-Zapirain, 2015) and adaptive filtering. Within these pipelines, Independent Component Analysis (ICA) and Principal Component Analysis (PCA) (Subasi and Gursoy, 2010) are fundamental techniques for decomposing complex, multi-channel EEG mixtures into highly interpretable and isolated components.

ICA de composes multi-channel scalp data into statistically independent, non-Gaussian components (Subasi and Gursoy, 2010; Jonna and Natarajan, 2025). This enables the isolation and removal of physiological artifacts such as eye blinks (EOG), lateral eye movements, muscle activity (EMG), and cardiac interference based on their distinct spatial topographies and temporal signatures. When combined with automated cleaning tools, ICA quickly pinpoints and removes eye blinks and sudden muscle noise. This makes the brainwave data much clearer before it is sent to the machine learning software. Besides just removing noise, ICA also helps separate mixed signals so researchers can study specific, meaningful brain patterns on their own.

Complementing this, PCA (Subasi and Gursoy, 2010) serves as an efficient mathematical framework for dimensionality reduction and noise suppression. Because EEG recordings are frequently high-dimensional, redundant, and highly correlated, PCA projects the data to retain the most significant variance while discarding components dominated by noise or irrelevant activity. In the preprocessing stage, PCA is often applied before ICA to reduce computational load and improve algorithmic stability by selecting only the principal components that capture meaningful structure. Furthermore, PCA is widely utilized in machine learning pipelines to compress features, which enhances classifier performance and mitigates overfitting. It also assists in microstate analysis by extracting dominant scalp topographies and simplifying global field power peaks before clustering. Together, ICA and PCA provide a robust foundation for simplifying EEG data while preserving its most informative features.

### 2.2 Public data repositories and clinical benchmarking frameworks

To assist future researchers, establish cross-site diagnostic robustness, and avoid overfitting stemming from subject-dependent validation protocols, models must be benchmarked using open-access clinical datasets. Table 2 outlines widely cited, publicly accessible EEG data repositories specifically dedicated to clinical disease classification and neurological pathology evaluation. Non-diagnostic repositories focused exclusively on healthy-subject motor imagery, affective computing, or active brain-computer interface (BCI) control have been excluded to maintain a strict focus on pathological disease diagnosis.

**TABLE 2:** Summary of publicly available open-access clinical EEG datasets for benchmarking neurological disease diagnostic models.

Dataset name	Dataset metrics	Disease type	Format and preprocessing	Limitations and known biases	Recommended validation strategy	Link
Kaggle/UCI Alzheimer’s EEG Dataset (Miltiadous et al., 2023)	Fs: 500 Hz 88 subjects (AD, FTD, Controls) Mean age: ∼65 years 19 channels	Neurodegenerative (Alzheimer’s/Dementia)	Format:.edf/OpenNeuro BIDS Preprocessing: 0.5–45 Hz band-pass filter; Artifact Subspace Reconstruction (ASR) + ICLabel-driven ICA.	Limited sample size per diagnostic cohort; lacks longitudinal behavioral metrics.	10-fold Subject-Independent (Group-level) split to avoid identity/biometric leakage.	OpenNeuro Repository (https://openneuro.org/datasets/ds004504/versions/1.0.9)
Helsinki Neonatal Seizure Dataset (Stevenson et al., 2018)	Fs: 256 Hz 79 term neonates (39 seizure, 22 seizure-free, 17 ambiguous) 19 channels	Neonatal Encephalopathy/Seizures	Format:.edf/.mat vectors Preprocessing: Consensus visual verification by 3 pediatric neurophysiologists. Continuous NICU monitoring.	High prevalence of environmental and motion artifacts typical of intensive care wards; highly skewed epoch count.	Leave-One-Subject-Out (LOSO) cross-validation to handle high inter-infant brain variability.	Zenodo Repository https://zenodo.org/records/2547147
CHB-MIT Scalp EEG Dataset (Shoeb, 2010)	Fs: 256 Hz 22 pediatric subjects 23 channels	Epilepsy	Format:.edf Preprocessing: Long-term clinical pediatric monitoring; continuous raw records.	Pediatric cohort bias; channel configurations vary slightly across a few subjects; extreme class imbalance.	Strict LOSO cross-validation to prevent subject-level data contamination.	PhysioNet Seizure Archive https://physionet.org/content/chbmit/1.0.0/
Bangalore EEG Epilepsy Dataset (BEED) (Najmusseher, 2024)	Fs: 256 Hz/512 Hz 80 adult subjects (ages 21–55; epileptic cohorts vs. healthy controls) 16 channels	Epilepsy	Format:.csv /.mat Preprocessing: Pre-segmented interictal spikes and baseline segments labeled by expert clinical epileptologists.	Pre-extracted static segments strip away the continuous, real-world temporal sequence context.	Pre-defined hold-out validation partitions to benchmark baseline ML/DL classifiers.	UCI Repository Gateway https://archive.ics.uci.edu/dataset/1134/beed:+bangalore+eeg+epilepsy+dataset
University of Bonn EEG Dataset (EEG-Dataset, n.d.)	Fs: 173.61 Hz 10 subjects (5 healthy, 5 with epilepsy) 1 channel	Epilepsy	Format: ASCII/Text Preprocessing: Pre-segmented, 23.6-second artifact-free epochs.	Highly artificial pre-cleaned segmenting completely removes real-world artifact handling context; extremely small subject pool.	Useful for initial pipeline testing, but highly prone to accuracy inflation; cross-dataset testing recommended.	Bonn Data Link https://www.kaggle.com/datasets/quands/eeg-dataset
Siena Scalp EEG Database (Detti, 2020)	Fs: 512 Hz 14 subjects with drug-resistant focal epilepsy 20–29 channels	Epilepsy	Format:.edf (PhysioNet) Preprocessing: Exact onset/offset seizure timestamps marked manually via video-EEG auditing.	Very small participant footprint poses an extreme risk of deep learning model overfitting; highly unbalanced time series.	Inter-subject stratified splits or strict K-fold LOSO.	PhysioNet Siena Archive https://physionet.org/content/siena-scalp-eeg/1.0.0/
PD-EEG: Parkinson’s Disease Dataset (Vered et al., 2026)	Fs: 512 Hz/1024 Hz 116 PD patients, 28 healthy controls 64 channels	Neurodegenerative (Parkinson’s Disease)	Format:.edf/BIDS standard Preprocessing: Linear notch filtering (50 Hz); artifact rejection via manual independent component scrubbing	Massive demographic class imbalance (116 patients vs. 28 controls).	Synthetic Minority Over-sampling Technique (SMOTE) applied strictly within training folds combined with LOSO.	OpenNeuro PD Portal https://openneuro.org/datasets/ds007526/versions/1.0.2
Mendeley IDD EEG Dataset (Sareen et al., 2020)	Fs: 128 Hz 14 participants (7 Intellectual/Developmental Disorder, 7 Controls) 14 channels	Intellectual and Developmental Disorders (IDD)	Format:.mat matrix Preprocessing: Raw data with minimal hardware bandpass filtering (0.2–45 Hz).	Lower signal-to-noise ratio (SNR) due to dry/saline consumer-grade hardware compared to clinical systems.	Subject-level validation to explicitly test model performance under lower-fidelity consumer arrays.	https://data.mendeley.com/datasets/fshy54ypyh/2
Sleep-EDF Database Expanded (Kemp et al., 2018)	Fs: 100 Hz 197 whole-night recordings from 80 subjects (including sleep-onset insomnia) 2–4 channels	Sleep Disorders/Somnology	Format:.edf (PhysioNet) Preprocessing: 30-second epoch intervals manually scored according to Rechtschaffen and Kales (R&K) patterns.	Sparse spatial footprint (2–4 channels) severely limits the application of advanced Spatial-CNN or graph topologies.	Pre-defined Subject-wise evaluation splits to mirror automated clinical diagnostic settings.	PhysioNet Sleep Hub https://physionet.org/content/sleep-edfx/1.0.0/
Temple University Hospital (TUH) EEG (Temple University EEG Corpus, n.d.)	Fs: Variable (250–512 Hz) 12,000+ patients; mixed clinical phenotypes and age representations Variable channels	Mixed Neurological/General Pathology	Format:.edf Preprocessing: Semi-automated annotation using NLP-parsed clinical EEG reports.	High real-world noise, varied hardware amplifiers, and non-standardized clinical protocol shifts across years.	Rigorous cross-dataset evaluation or pre-defined, standardized train/test splits.	TUH Project Index https://isip.piconepress.com/projects/tuh_eeg/

The table outlines sample sizes, recording parameters (sampling frequency and channels), diagnostic target categories, preprocessing pipelines, known clinical biases, and recommended subject-independent validation protocols to prevent data leakage. Technical and recording terms: Fs, Sampling Frequency (Hertz); Channels, Scalp Electrode Channels; BIDS, Brain Imaging Data Structure; EDF, European Data Format; ASCII, American Standard Code for Information Interchange; SNR, Signal-to-Noise Ratio. Pathologies and cohorts, AD, Alzheimer’s Disease; FTD, Frontotemporal Dementia; PD, Parkinson’s Disease; IDD, Intellectual and Developmental Disorders; NICU, Neonatal Intensive Care Unit; HC, Healthy Controls. Preprocessing and algorithms, ICA, Independent Component Analysis; ASR, Artifact Subspace Reconstruction; ICLabel, Automated Component Classification Tool; SMOTE, Synthetic Minority Over-sampling Technique. Validation protocols: LOSO, Leave-One-Subject-Out Cross-Validation (a strict subject-independent validation strategy holding out all data of a single patient during training to prevent identity and biometric data leakage across folds).

### 2.3 Methods for EEG feature extraction

EEG feature extraction is a critical step that transforms raw, noisy brain signals into meaningful representations that can be used for interpretation, classification, or diagnostic modeling (Siuly et al., 2016). Because EEG signals are nonstationary, nonlinear, and highly heterogeneous across individuals and tasks, extracting informative features requires techniques from multiple domains, ranging from simple temporal measures to advanced spatial and nonlinear dynamics. Each feature domain captures distinct aspects of neural activity, such as amplitude changes, oscillatory patterns, transient events, microstate dynamics providing diverse computer-based metrics that help to make diagnostic models highly accurate and objective.

#### 2.3.1 Mathematical feature domains

##### 2.3.1.1 Time-domain features

Time-domain features remain a foundational category in EEG analysis, directly characterizing amplitude variations and waveform morphology (Geethanjali et al., 2012). By utilizing measures such as statistical moments, peak-to-peak values, root mean square (RMS), and Hjorth parameters, these methods effectively capture the raw temporal dynamics of the signal (Ceolini et al., 2020). Furthermore, recent frameworks demonstrate that when optimized time-domain metrics are paired with gradient-boosted ensemble classifiers, the resulting classifiers exhibit surprising robustness, maintaining high multi-state classification accuracy even at low signal-to-noise ratios (SNRs) (Nassir et al., 2025). However, despite these algorithmic efficiencies, time-domain metrics remain highly sensitive to transient ocular or muscular artifacts and fundamentally lack the capacity to isolate oscillatory, frequency-specific neural processes.

##### 2.3.1.2 Frequency-domain features

Frequency-domain features address these limitations by quantifying the power distribution across canonical EEG frequency bands. Beyond traditional metrics like Fast Fourier Transform (FFT), Welch’s periodogram (Hazarika et al., 1997), and autoregressive modeling, contemporary machine learning pipelines increasingly leverage advanced spectral descriptors including spectral entropy, spectral flatness, and the log-linear spectral slope (1/f broadband dynamics). These features offer robust, highly interpretable links to distinct neurophysiological states, such as focal delta or theta slowing indicative of structural abnormalities or localized ischemia (Dockree et al., 2007), or alpha rhythm degradation reflecting progressive neurodegeneration (Trammell et al., 2017), or broadband shifts indicative of neurological aging and cortical excitation-inhibition balance. Nonetheless, because standard frequency-domain analysis relies heavily on the assumption of signal stationarity within fixed windows, these methods inherently struggle to capture rapid, non-stationary neural dynamics, acute state transitions, or micro-scale transient phenomena.

##### 2.3.1.3 Time-frequency and nonlinear features

To overcome the constraints of isolated temporal or spectral analyses, time-frequency feature extraction methods such as wavelet (Jacob et al., 2018b), Stockwell and Hilbert-Huang transforms (Huang, 2014), alongside empirical (EMD) and variational mode decomposition (VMD) (Kaur et al., 2021), provide simultaneous temporal and spectral resolution. These advanced representations excel at isolating nonstationary neural events such as sleep spindles, epileptic spikes, and task-related oscillatory bursts; however, they are computationally demanding, require careful parameter tuning, and methods like EMD (Riaz et al., 2015) can suffer from mode mixing and susceptibility to noise. Complementing these approaches, nonlinear feature extraction captures the inherently complex, chaotic (Jacob et al., 2018a,^2021^), and dynamical characteristics of EEG signals that elude traditional linear methods. This domain leverages entropy-based measures (sample, approximate, permutation, fuzzy, and multiscale entropy) (Jacob and Nair, 2019) to quantify the irregularity of neural activity, alongside fractal and scaling features including Higuchi and Katz fractal dimensions (Jacob et al., 2019; Wijayanto et al., 2019), the Hurst exponent (Geng et al., 2011), and detrended fluctuation analysis to characterize self-similarity and long-range correlations. Furthermore, chaos-theoretic metrics like the largest Lyapunov exponent (Krystal et al., 1997), correlation dimension, and phase-space reconstruction features map underlying brain system dynamics, while complexity indices such as Lempel Ziv (Ibáñez-Molina et al., 2015), Rényi (Guisande and Montani, 2024), and approximations of Kolmogorov complexity (Arunkumar et al., 2016) capture information richness and structural variability. When paired with recurrence quantification measures (recurrence rate, determinism, and laminarity) derived from recurrence plots (Hazarika et al., 1997; Chandrasekharan et al., 2024), these combined frameworks reveal hidden temporal patterns and state transitions. Together, these nonlinear metrics offer exceptional sensitivity to subtle neural alterations in disorders such as epilepsy (Jacob et al., 2016), Alzheimer’s disease (Chen et al., 2023), and depression, though they remain computationally intensive, sensitive to noise, and challenging to interpret clinically.

##### 2.3.1.4 Spatial and connectivity features

Finally, spatial, connectivity, and graph-based features exploit the multichannel structure of EEG to map and quantify complex interactions between distinct brain regions. Functional and directed connectivity measures such as coherence, phase-locking value, Granger causality, transfer entropy, and the weighted phase lag index enable the deep characterization of large-scale brain networks. When paired with graph-theoretical metrics (Demir et al., 2021), these approaches provide critical insights into network topology, information routing, and global efficiency, making them powerful assets for studying neurological disorders, cognitive processes, and high-dimensional machine learning applications. However, these methods depend heavily on high-quality preprocessing, remain computationally intensive, and can be severely confounded by volume conduction and reference electrode effects. Despite these challenges, integrating spatial and connectivity-based features with traditional domains yields the most comprehensive, multidimensional representation of brain dynamics, forming a robust and advanced framework for state-of-the-art EEG feature extraction (Figure 4).

**FIGURE 4:**
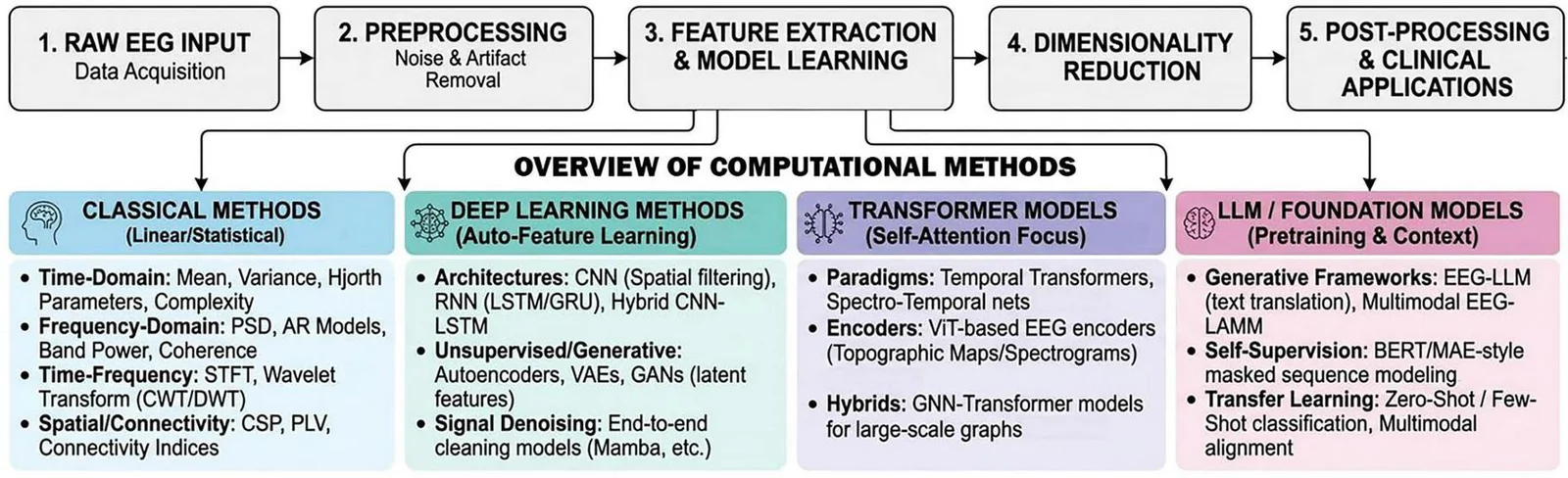
Comprehensive framework of EEG feature extraction methodologies.

#### 2.3.2 Deep learning data-driven features

In addition to classical feature domains, recent advancements in deep learning have introduced powerful data-driven feature extraction approaches that automatically learn discriminative representations directly from raw or lightly preprocessed EEG signals. Convolutional neural networks (CNNs) (Mao et al., 2020) hierarchically capture spatial patterns, frequency-specific structures, and local temporal dependencies without manual feature engineering, while recurrent architectures such as Long Short-Term Memories (LSTM) (Li K. et al., 2024) and Gated Recurrent Units (GRU) (Wang et al., 2022) effectively model long-range temporal dynamics and nonstationary neural fluctuations. Building upon these foundations, transformer-based models (Ghous et al., 2025) employ self-attention mechanisms to learn global relationships across channels and time, enabling them to identify context-dependent interactions and cross frequency couplings often missed by classical methods. Most recently, large language model inspired architectures treat EEG sequences as structured temporal “tokens,” allowing the deployment of self-supervised pretraining strategies, cross-modal embeddings, and zero or few-shot learning to drastically improve generalization across heterogeneous subjects and tasks. Although these deep architectures offer exceptional flexibility, nonlinear modeling capacity, and state-of-the-art performance, they are constrained by high data volume requirements, substantial computational overhead, and persistent challenges in clinical interpretability. Performance liabilities notwithstanding, these foundation models represent the modern frontier of EEG analytics, complementing traditional feature engineering to enable highly automated, robust, and scalable neural decoding.

### 2.4 Subspace optimization and dimensionality reduction

Feature selection and dimensionality reduction are critical bottlenecks in the EEG analytical pipeline. High-density recordings yield massive, high-dimensional datasets typically rife with multi-channel redundancy, irrelevant features, and physiological noise (see Figure 5). Implementing subspace optimization aims to isolate the most informative neural representations while discarding distracting or null components. This targeted compression prevents model overfitting, lowers computing demands, and makes automated disease diagnosis much more reliable (Lan, 2011).

**FIGURE 5:**
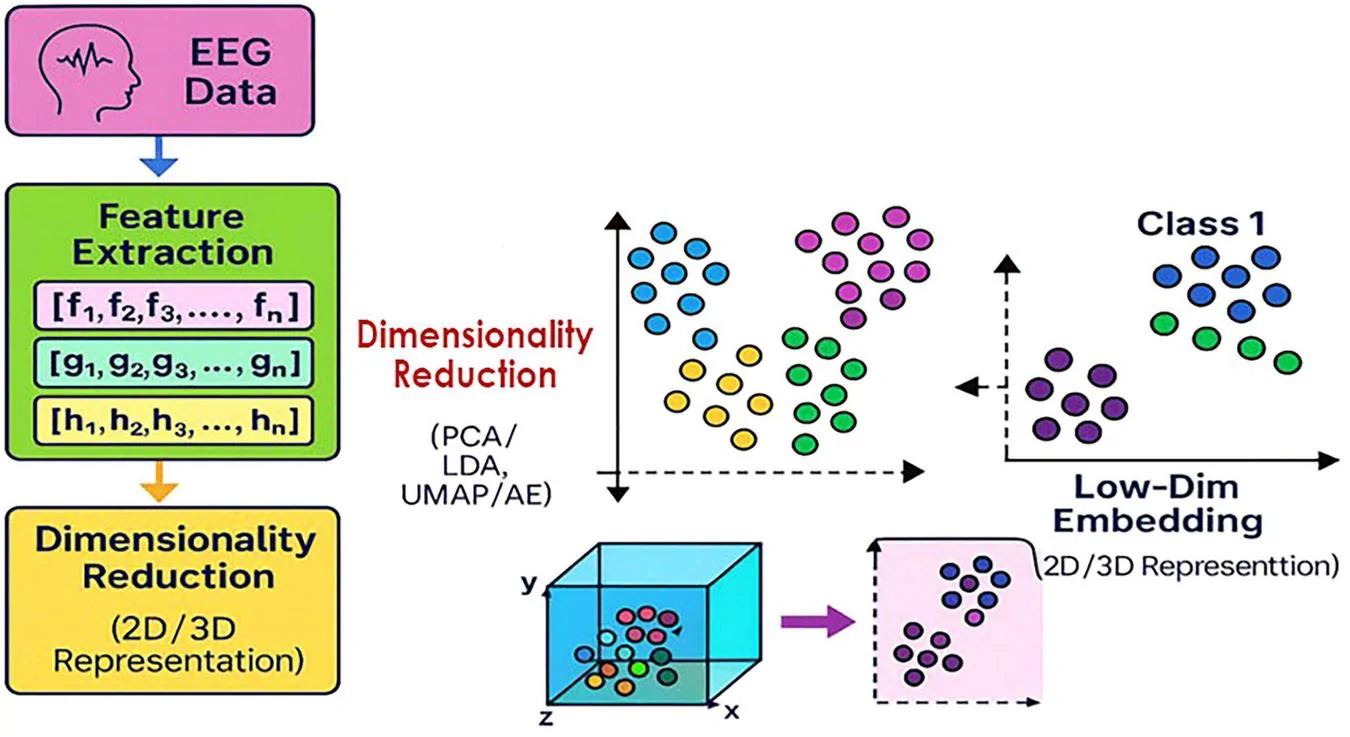
Schematic diagram showing the EEG feature extraction stages and subsequent dimensionality-reduction process.

#### 2.4.1 Feature selection strategies

Feature selection identifies the most highly discriminative subsets from an existing feature array. Traditional pipelines rely heavily on statistical and mathematical filters such as mutual information, max-relevance min-redundancy (mRMR) (Hui et al., 2024), recursive feature elimination (RFE), and Least Absolute Shrinkage and Selection Operator (LASSO) regularization. (Sreedhar et al., 2025). To manage increasingly complex, high-density EEG configurations, modern paradigms (2023–2026) have introduced advanced strategies. These include graph-based feature ranking, multi-objective evolutionary search algorithms, stability selection via repeated subsampling, and transformer-derived attention mapping that dynamically surfaces critical neural markers. Collectively, these modern selection mechanics provide superior noise immunity and maximize spatial-temporal feature efficiency.

#### 2.4.2 Linear and non-linear dimensionality reduction

Rather than selecting subsets, dimensionality reduction projects high-dimensional EEG feature spaces into compact, lower-dimensional latent representations. Classical linear approaches like Principal Component Analysis (PCA) and Independent Component Analysis (ICA) (Subasi and Gursoy, 2010) offer orthogonal and statistically independent projections, respectively. While PCA compresses data by maximizing descending variance and suppressing redundant channel noise, ICA decomposes multi-channel mixtures to isolate distinct neural sources from non-neural artifacts (Mannan et al., 2018; Lotte and Congedo, 2016). Where linear projections fail to map complex manifold structures, non-linear alternatives like t-Distributed Stochastic Neighbor Embedding (t-SNE) (Li et al., 2016), Isomap, and modern Uniform Manifold Approximation and Projection (UMAP) (Yue et al., 2025) excel. These are further complemented by deep-learning architectures, including variational autoencoders (VAEs), contrastive self-supervised encoders, and transformer-based latent compressors, which yield highly dense embeddings that preserve underlying neurophysiological topologies.

### 2.5 Classification, regression, and post-processing pipelines

#### 2.5.1 Classification and regression architectures

Optimized, low-dimensional EEG feature representations serve as direct inputs for supervised downstream learning tasks. For categorical classification, such as mapping or grading discrete disease pathologies (see Figure 6), interpretable classical architectures like Support Vector Machines (SVM) (Saadoon et al., 2025), Linear Discriminant Analysis (LDA) (Subasi and Gursoy, 2010), k-Nearest Neighbors (k-NN) (Laaksonen and Oja, 1996), and Random Forest or Gradient Boosting (Callens et al., 2020) ensembles remain foundational. Concurrently, deep learning frameworks such as CNNs (Mao et al., 2020), recurrent networks (LSTM/GRU) (Rivas et al., 2025), hybrid CNN-RNN configurations (Shi et al., 2019), and self-attention transformers (Lee and Lee, 2022) handle complex, non-linear spatiotemporal dependencies. For continuous tracking regression tasks (e.g., estimating mental workload, stress index, or clinical severity), pipelines deploy regularized linear models and neural regressors (Zhang and O’Donnell, 2020). These networks are bounded by strict validation bounds (e.g., k-fold, subject-independent validation) and evaluated using robust parameters (*R*^2^, Mean Absolute Error [MAE], Root Mean Squared Error [RMSE] for regression; F1-score, Cohen’s kappa, and AUC for classification) (Oliveira et al., 2016; Sharpee et al., 2016).

**FIGURE 6:**
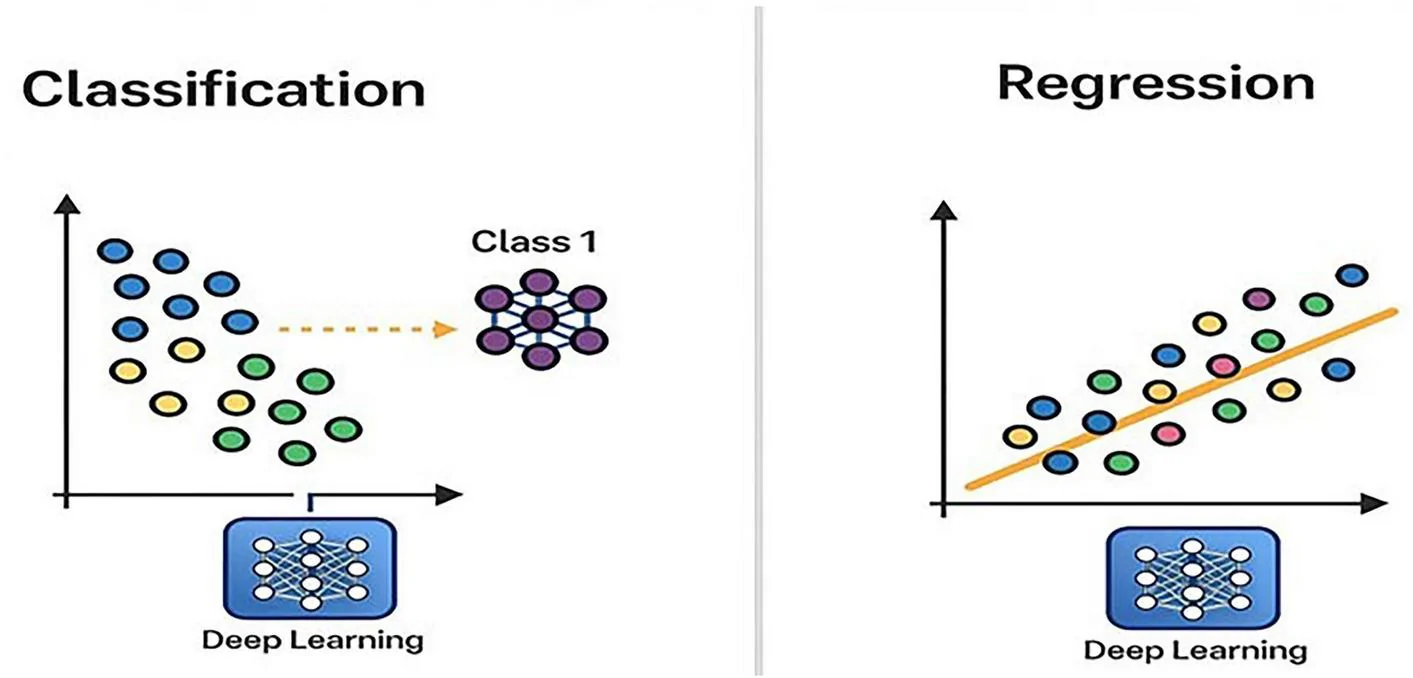
Schematic representation of supervised learning workflows in EEG analysis: Classification and regression.

#### 2.5.2 Post-processing, interpretability, and clinical translation

The final phase stabilizes model predictions to ensure real-world utility. Post-processing refines and validates outputs, often employing temporal smoothing windows, majority voting, or statistical significance testing, to mitigate prediction instability caused by inter-subject variability (Baltatzis et al., 2017). To mitigate the “black box” liability of deep learning, model interpretation strategies utilize attention weights, feature-importance metrics like SHapley Additive exPlanation (SHAP) or Local Interpretable Model-agnostic Explanations (LIME), and saliency maps. These methods map model decisions back to neurophysiologically meaningful features, including specific EEG channels, critical temporal windows, or localized frequency bands (e.g., alpha/beta shifts or microstate transitions). When translated into clinical and assistive environments, these explainable frameworks provide robust diagnostic biomarkers for conditions such as epilepsy (Acharya et al., 2013), mild cognitive impairment, Alzheimer’s disease, depression, and Attention-Deficit/Hyperactivity Disorder (ADHD) (Bansal et al., 2025), while providing dependable motor imagery and workload decoding for neurological rehabilitation and assistive technologies.

## 3 Deep learning architectures for EEG processing

Deep learning (DL) models have emerged as the dominant paradigm for electroencephalography (EEG) signal cleaning due to their unique capacity to learn complex, non-linear mappings directly from raw data. In algorithmic denoising, an observed, artifact-contaminated EEG segment Y is conventionally modeled as the additive combination of the underlying, uncorrupted neural signal X and an overlapping noise component Z (encompassing ocular, muscular, cardiac, or instrumental disturbances) (Merlin Praveena et al., 2022):


Y=X+Z

The main goal of the deep learning denoising framework f(Y) is to produce a clean signal estimate that closely matches the real signal X in structure, phase, and frequency (refer to Figure 7a). This parameter optimization is driven by minimizing an objective loss function, most frequently the Mean Squared Error (MSE) (Raj et al., 2025)


$$£=1/n\,\overset{n}{\underset{i=1}{\sum }}\left({\left(f_{\theta }\,\left(y_{i}\right)-{\text{x}}_{i}\right)}^{2}\right)$$

**FIGURE 7:**
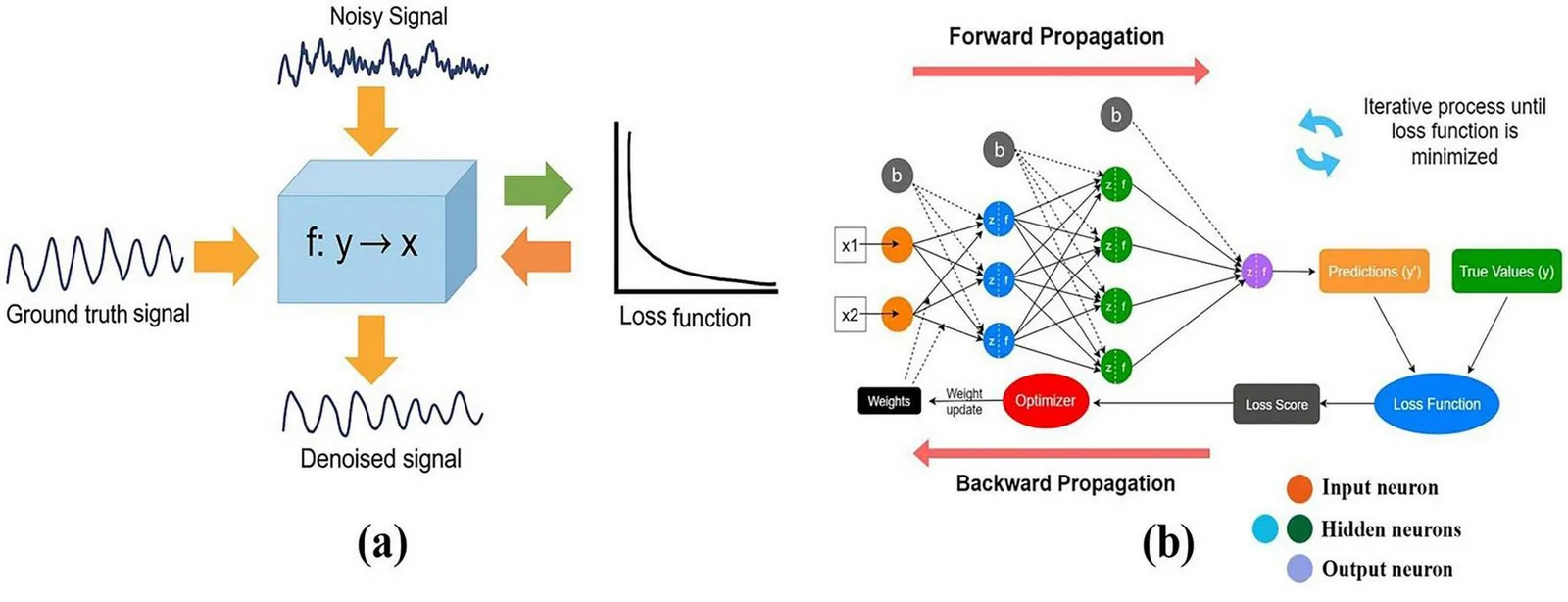
**(a)** Depicts the nonlinear mapping f, which takes the noisy EEG input y and produces an estimate of the clean signal x. **(b**) Illustrates how the network processes the input during forward propagation and updates its parameters through backward propagation.

where n represents the total number of temporal samples within the EEG segment. While individual architectures leverage distinct inductive biases to process these signals, they all share a baseline execution cycle governed by forward propagation, loss calculation, and backward error propagation (Acharya et al., 2013).

During the forward propagation phase, the input tensor passes through deep neural layers, where each neuron processes the signal by multiplying the input vectors (*x_i_*) by learned weights (*w_i_*) and adding a bias term (b) (Wong et al., 2018)


$$Z=f\,\left(\underset{\text{i}}{\sum }{\text{x}}_{\text{i}}\,{\text{w}}_{\text{i}}+\text{b}\right)$$

In EEG processing, these weights functions of adaptive filters are (Jafarifarmand and Badamchizadeh, 2013) optimized to identify temporal and spatial neural patterns, while the bias term stabilizes neuron activation under low-amplitude or highly noisy conditions. As the signal travels across layers, non-linear activation functions [e.g., Rectified Linear Unit (ReLU) or hyperbolic tangent (tanh)] help the network learn the complex boundaries separating real brain activity from noise (refer to Figure 7b).

Backward propagation begins immediately after the network generates its denoised output and measures its discrepancy against the ground truth. This error is propagated backwards through the network to calculate gradients using optimization algorithms like Adam (Fahrenfort et al., 2018), RMSProp (Zou et al., 2019), or Stochastic Gradient Descent (SGD)(Bottou, 2012), updating the weights and biases to reduce overall error. During this learning cycle, additional techniques such as regularization (L2, dropout), batch normalization, skip connections (as in U-Net) (Hossain et al., 2023), and auxiliary spectral loss constraints help the model prevent overfitting and preserve essential brain rhythms across varying clinical segments.

### 3.1 Convolutional neural network (CNN)

In EEG analysis, Convolutional Neural Networks (CNNs) (Mao et al., 2020) treat multi-channel time-series data as shifting spatial-temporal grids. Rather than relying on manual feature engineering, 1D convolution layers slide small learnable filters along the time axis to automatically extract shift-invariant local features like transient epileptic spikes (Khademi et al., 2022), rhythmic oscillations, or abrupt high-amplitude ocular deflections. Subsequent non-linear activations (primarily ReLU) model complex brain dynamics, while pooling layers systematically downsample the feature maps to retain only the most informative components. The learned features are ultimately combined through fully connected layers to generate a refined neural estimate or classify abstract representations related to distinct brain states (Figure 8; Chaudary et al., 2025).

*Input → Convolution → ReLU → Pooling → Convolution → ReLU → Pooling → Flatten → Fully Connected → Output*

**FIGURE 8:**
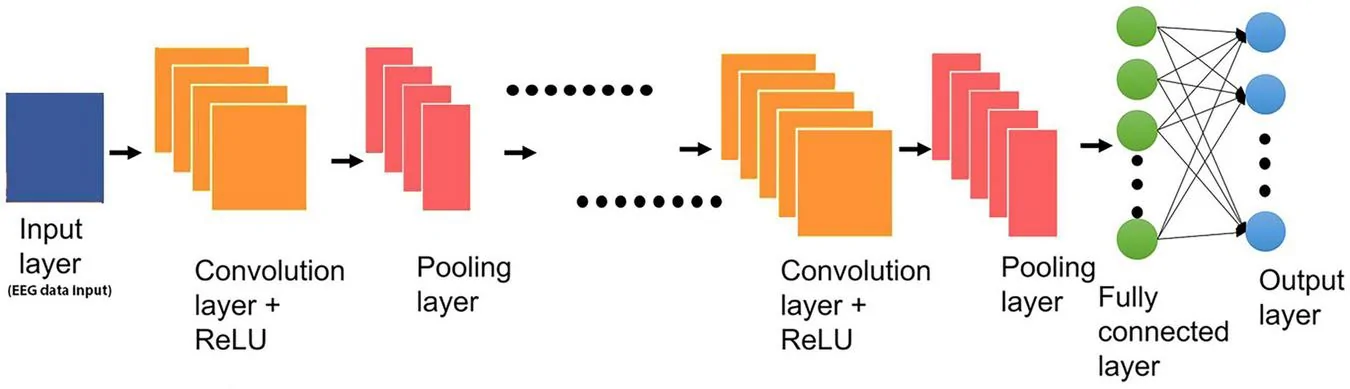
Basic architecture of a 1-D convolutional neural network.

The primary strength of CNNs lies in their exceptional capacity for isolating local, high-amplitude, and short-duration artifacts, making them the standard choice for handling EOG and EMG bursts. In medical software, this precise cleaning isolates noise to its exact moment in time, ensuring that brief, critical signs of disease like the sudden electrical spikes that signal an epileptic seizure are protected and not accidentally erased during automated screening. Regarding computational complexity, CNNs require lower overhead compared to sequential models; their highly parallelizable structure allows fast execution times, making them uniquely suited for low-latency bedside patient warning architectures (Pathak and Kashyap, 2022).

However, their major methodological limitation arises from the inherently localized nature of standard 1D convolutions, they fail to adequately model long-range temporal context and do not naturally capture complex spatial relationships across physically non-contiguous EEG channels (Xiong, 2023). Furthermore, standard CNN configurations optimized purely via MSE loss run a high risk of generating overly smooth outputs that can blur vital micro-amplitude neural information.

In comparative benchmark studies, 1D-CNNs and spatial-temporal variants (such as EEGNet) consistently demonstrate a strong balance between feature learning capacity and computational efficiency. On benchmark datasets like CHB-MIT and TUH EEG, CNN-based frameworks report classification accuracies ranging between 91.5 and 96.8%, compared to traditional SVM or Random Forest models which yield 84.0–89.5% on identical raw inputs (Mutlu et al., 2025). However, this gain in accuracy requires substantial training data and computational resources. On small datasets (e.g., < 50 subjects), conventional ML approaches paired with domain-specific feature extraction (such as DWT or Hjorth parameters) frequently match or outperform shallow CNN architectures while avoiding the risk of overfitting (Alqudah and Moussavi, 2025).

### 3.2 Recurrent neural networks (RNNs, LSTMs, and GRUs)

Recurrent Neural Networks (RNNs) (Gorur and Eraslan, 2022) are explicitly designed to handle sequential data, making them mathematically tailored for the time-varying, dynamic nature of continuous EEG signals. Unlike CNNs that analyze local windows in isolation, RNNs inherently maintain an ongoing hidden state (*h_t_*) that carries historical context from all previous time steps.

At each time instant t, the hidden state is updated conditionally based on the current EEG input (*x_t_*) and the previous state (*h*_*t–1*_).


$$h_{t}=f\,\left(W_{x\,h}\,x_{i}+W_{h\,h}\,h_{t-1}+b_{h}\right)$$

where *W*_*xh*_ and *W*_*hh*_ are weight matrices, *b_h_*is a bias term, and *f*(.) is a nonlinear activation function such as tanh or ReLU (Figure 9). The output y_*t*_ is then computed as


$$y_{t}=g\,\left(W_{h\,y}\,h_{t}+b_{y}\right)$$

**FIGURE 9:**
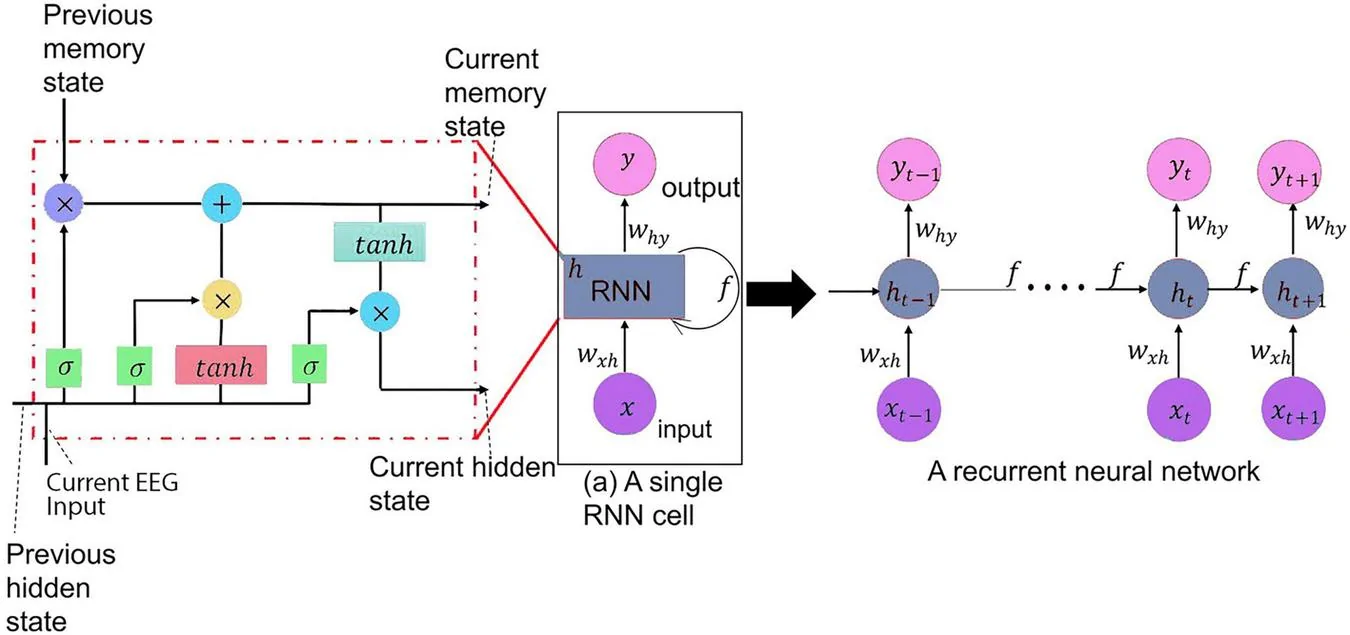
Architecture of an LSTM-based recurrent neural network. (Left) Detailed internal cell architecture handling memory and hidden states. (Right) Sequential unrolling of the recurrent network across time steps.

allowing the network to learn temporal patterns across EEG samples.

In terms of denoising suitability, RNNs are highly effective for isolating continuous, low-frequency, slow-moving physiological drift, such as respiratory artifacts, thermal sweat drift, or repetitive Electrocardiogram (ECG) pulse interference (Chaudary et al., 2025). This temporal effectiveness stems from their specific temporal tracking capabilities. However, their operational and computational complexity is inherently high; because RNNs process sequential data strictly sample-by-sample, they prevent efficient GPU parallelization (Peng et al., 2026), which leads to significantly longer training cycles and higher deployment latency.

Methodologically, standard RNNs suffer from vanishing and exploding gradients over long EEG sequences, and they cannot naturally map the 3D spatial layout of the scalp. While advanced gating architectures like LSTMs (Kashefi Amiri et al., 2025) and GRUs resolve these gradient issues, their numerous internal hyperparameters significantly increase the risk of overfitting on small datasets. Mechanistically, because every output step is chained to past context, a single high-amplitude movement artifact can completely derail the network’s hidden state trajectory, causing a cascading wave of distortion across subsequent samples. Furthermore, because standalone recurrent layers are blind to physical electrode locations, they struggle to resolve multi-channel spatial noise. To overcome these limitations, modern pipelines rarely use standalone recurrent frameworks, favoring hybrid CNN–RNN (Raj et al., 2025) or CNN–LSTM (Patel et al., 2025) architectures where convolutional layers extract spatial features before sequential temporal tracking.

### 3.3 Autoencoders (AEs)

Autoencoders are data-specific, self-supervised models whose primary inductive bias is based on architectural compression and reconstruction (Abdi-Sargezeh et al., 2025). The network is divided into three parts: an Encoder that maps a high-dimensional noisy EEG segment (X) into a compact, low-dimensional Bottleneck (latent) layer *h = f(X) = φ(W_*e*_X + b_*e*_)*, and a Decoder that reconstructs the signal back into a clean estimate *X̂ = g(h) = ψ(W_*d*_ h + b_*d*_)* (Figure 10; Bansal et al., 2025).

**FIGURE 10:**
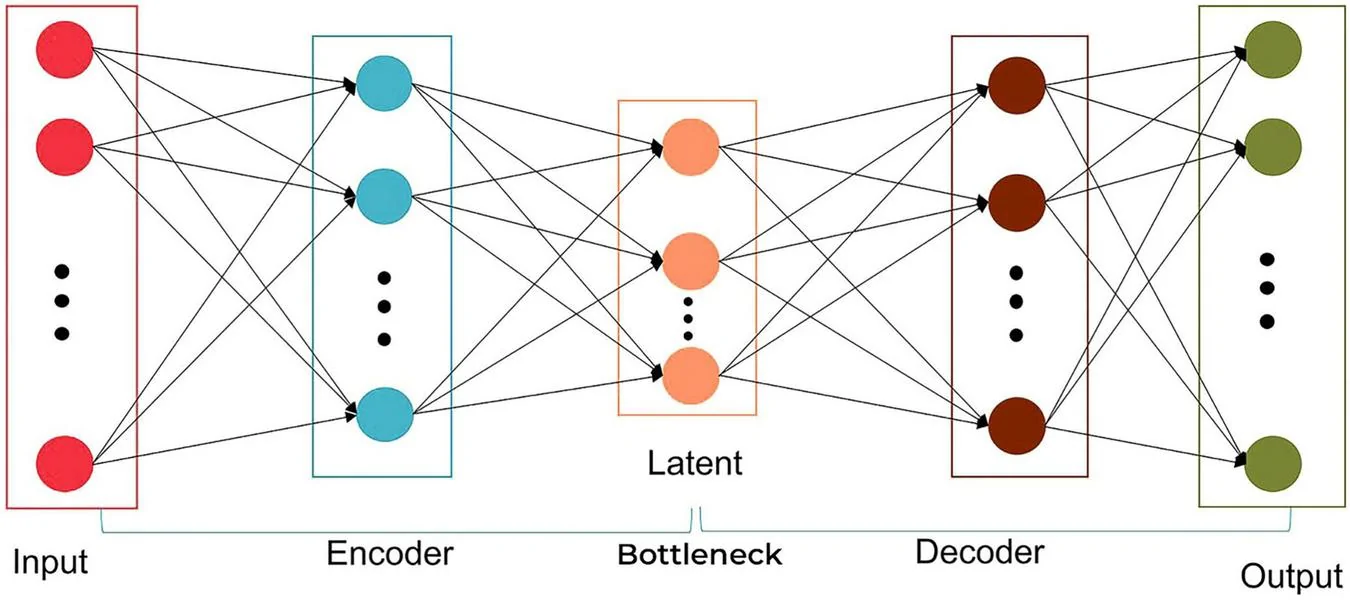
Basic architecture of an autoencoder network. The encoder compresses the input data into a lower-dimensional latent space (bottleneck), while the decoder reconstructs the original input data at the output layer.

By forcing the contaminated signal through a tight information bottleneck, the network is compelled to suppress redundant noise and stochastic artifacts while retaining stable underlying neural components. Specialized variants enforce unique structural constraints to improve denoising performance:

#### 3.3.1 Denoising autoencoders (DAEs)

Trained by deliberately corrupting the input with artificial noise while forcing the network to reconstruct the original, clean signal. This corrupt-then-reconstruct process compels the network to learn noise-invariant feature mappings rather than trivial identity functions, making it robust to real-world artifacts at inference time (Vincent et al., 2008; Koren et al., 2018).

#### 3.3.2 Sparse autoencoders

Impose a sparsity penalty on the latent layer’s activations (e.g., an L1 or KL-divergence-based constraint), driving most latent neurons toward zero for any given input. Only a small subset of neurons remain active at a time, encouraging each to specialize in a distinct, high-value feature helping the network isolate specific neural firing patterns rather than encoding redundant, overlapping representations (Pach et al., 2026).

#### 3.3.3 Variational autoencoders (VAEs)

Model the latent space as a continuous probability distribution (typically Gaussian) (Yeoh et al., 2026) regulated by a Kullback–Leibler (KL) divergence (Naidji et al., 2026) term to ensure smooth, well-organized reconstructions:


$${ℒ}_{V\,A\,E}\left(\theta ,\varphi ;;x\right)={𝔼}_{q_{\varphi \,\left(z/x\right)}}\left[logp_{\theta }\left(x/z\right)\right]-\beta D_{K\,L}\left(q_{\varphi \,\left(z/x\right)}∥p\left(z\right)\right)$$

Autoencoders demonstrate strong denoising suitability, being highly effective for blind source separation, baseline wander elimination, and automated artifact blanking across homogeneous baseline brain states. The principal methodological limitation of autoencoders is that they are extremely data-specific; variations between training and deployment data distributions severely degrade reconstruction accuracy. Because autoencoders are inherently lossy, an over-constrained bottleneck layer routinely suppresses fine-grained, low-amplitude neurogenic details, leading to overly smooth outputs that can mask subtle diagnostic markers. Furthermore, the compressed features captured within the latent space are classified high-level representations related to distinct brain states (Wang et al., 2026).

### 3.4 Generative adversarial networks (GANs)

Generative Adversarial Networks (GANs) formulate the problem of EEG artifact removal as a zero-sum, competitive game between two distinct components (Tibermacine et al., 2025): a *Generator (G)* and a *Discriminator (D)*. The Generator, typically built using convolutional layers, autoencoders, or residual blocks, learns to transform artifact-contaminated inputs (*X*_*raw*_) into clean neural estimates (Li X. et al., 2026). Concurrently, the Discriminator uses deep convolutional layers to evaluate whether the generator’s output looks like a real, pristine EEG signal (X) (Figure 11). The adversarial loop optimizes a minimax objective function (Liang et al., 2026)


$$\min_{G}\max_{D}V\,\left(D,G\right)={𝔼}_{x∼p_{c\,l\,e\,a\,n}\,\left(x\right)}\,\left[\log D\,\left(x\right)\right]+$$

$${𝔼}_{\tilde{x}∼p_{n\,o\,i\,s\,y}\,\left(\tilde{x}\right)}\,\left[\log\left(1-D\,\left(G\,\left(\tilde{x}\right)\right)\right)\right]$$

**FIGURE 11:**
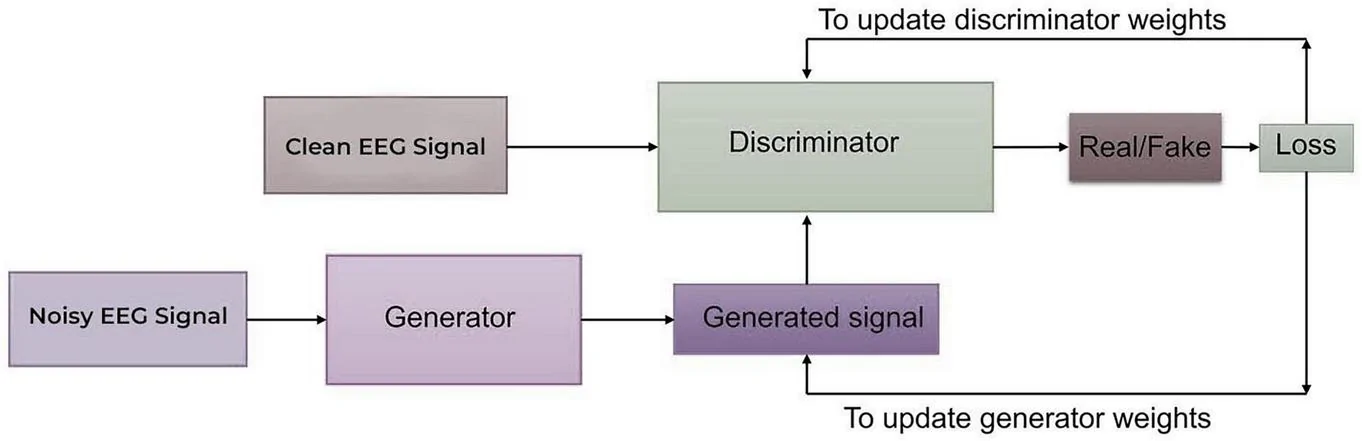
The basic architecture of generative adversarial networks for EEG signal processing, including data flow for generator and discriminator updates, clean and noisy signal paths, and the loss evaluation.

To prevent the model from drifting, a combined loss strategy is deployed where an adversarial loss ensures realism while a reconstruction loss (MSE or MAE) (Lee et al., 2024) enforces structural consistency. Advanced iterations incorporate structural improvements, such as CycleGANs (Bouallegue and Djemal, 2020), which use a cycle-consistency principle to denoise unlabeled or real-world datasets where paired clean data is completely unavailable, and Wasserstein GANs (WGANs) (Dong et al., 2023), which replace standard probabilities with Wasserstein distance metrics to stabilize training.

Functionally, GANs are excellent at keeping the structure of multi-channel EEG signals intact and creating clean, high-quality estimates from very noisy data. However, this comes at the cost of massive computational complexity, requiring heavy GPU power and constant, delicate adjustments to keep the training process stable. Because the generator’s only goal is to trick the discriminator rather than follow real biological rules, it is highly prone to “mode collapse,” a failure mode in which the generator repeatedly outputs the same wave pattern. From a medical standpoint, an over-aggressive model can easily erase tiny, critical signs of disease or accidentally fabricate fake brain waves that look real but are completely artificial. Because true, uncorrupted human brain signals are rarely available as ground-truth supervision in real clinical settings, finding a way to prove whether a cleaned signal is real or faked remains a major roadblock to using GANs in hospitals (Li X. et al., 2026).

### 3.5 Transformers

Transformers bypass recurrent loops entirely, utilizing an architecture based on Self-Attention mechanisms to dynamically weight and focus on different temporal segments of an input sequence (He et al., 2024). Because EEG signals are non-stationary, this selective focus allows the network to adapt to changing signal traits much more effectively than traditional deep networks. The architecture is composed of stacked encoder and decoder layers (Figure 12).

**FIGURE 12:**
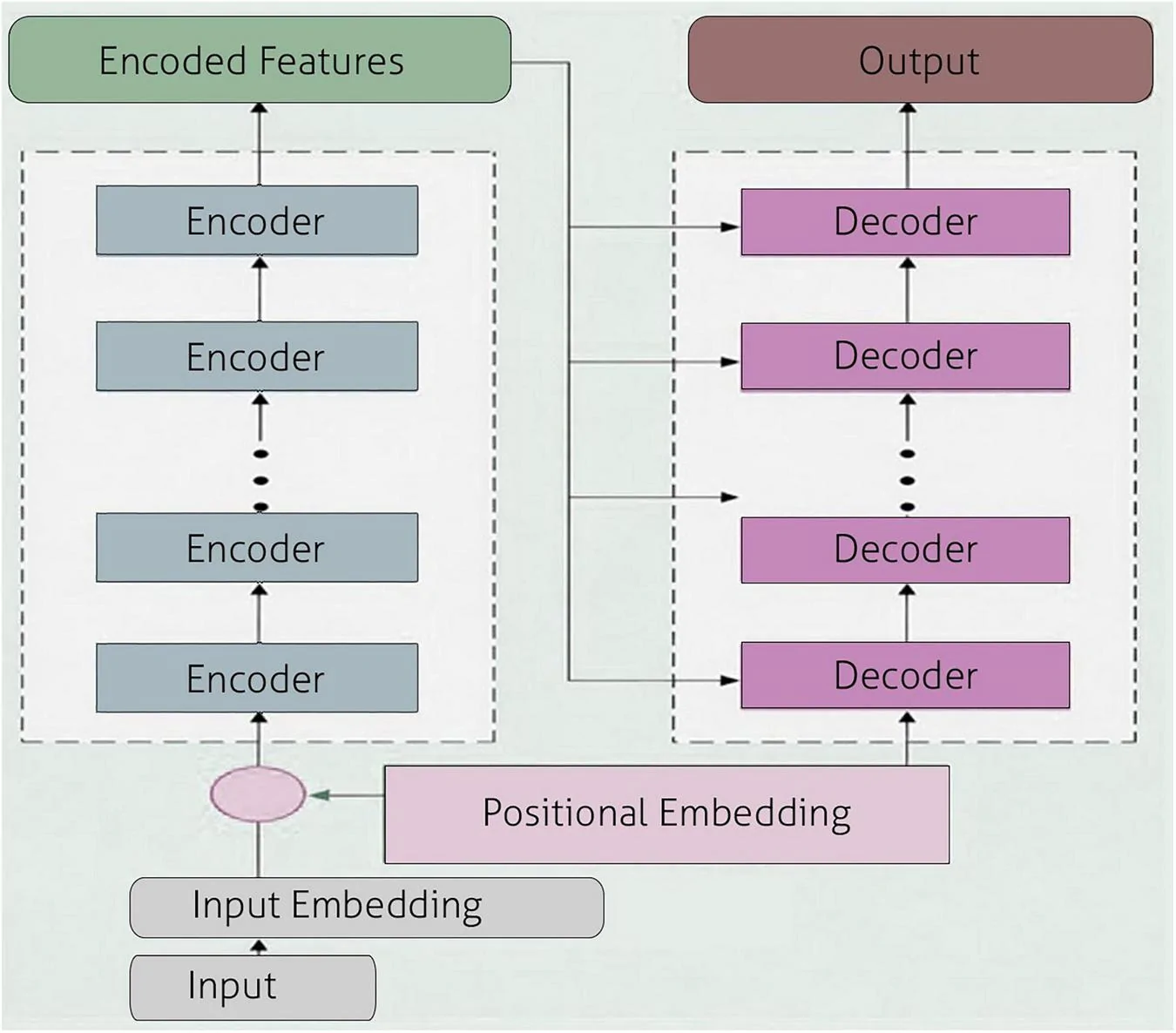
The basic architecture of transformers, showing how the input signal is combined with time-order information (positional embedding) and passed through stacked encoder and decoder blocks to produce the final output.

Because transformers do not inherently encode temporal order, sinusoidal *Positional Encodings* are added directly to the high-dimensional feature-embedded representations *E = XW_*e*_+ b_*e*_* prior to the encoder stage (Ghous et al., 2025). For each attention head, features are split into Query (Q), Key (K), and Value (V) matrices to calculate Scaled Dot-Product Attention:


$$A\,t\,t\,e\,n\,t\,i\,o\,n\,\left(Q,K,V\right)=s\,o\,f\,t\,m\,a\,x\,\left(\frac{Q\,K^{T}}{\sqrt{d_{k}}}\right)\,V$$

This allows the network to efficiently model complex temporal dependencies across non-contiguous windows. Specialized variants include *Time-series transformers* that process window-based sequences, Vision transformers (ViTs) (Zhou et al., 2026) that view 2D time-frequency spectrogram maps or topographic layouts as image-like inputs, and Spectral transformers (De la Rosa et al., 2026) that target noise signatures directly within the frequency domain.

Functionally, self-attention allows Transformers to excel at capturing long-term timing patterns, tracking unpredictable noise bursts, and analyzing how different EEG channels interact with each other. However, because the computational workload grows quadratically with the length of the signal, these models require massive computer memory and long training times when handling high-density, multi-channel data (Bollens et al., 2025). Transformers do not naturally understand continuous time sequences or local spatial arrangements. Because of this, they need exceptionally large datasets or extensive data augmentation to learn effectively and avoid simply memorizing the training data. While shortcuts like downsampling or adding convolutional layers can shorten the data sequence, these modifications carry a high risk of erasing tiny, crucial medical details. Finally, the dense layers of attention maps function as complex “black boxes,” making them much harder for clinicians to interpret and trust compared to traditional filtering methods (Ghous et al., 2025).

### 3.6 Large language model (LLM) inspired approaches

Large language models (LLMs) (Chandrasekharan and Jacob, 2025), though originally engineered for natural language processing, are increasingly being adapted for advanced generative neuroinformatics and clinical decision support. In these frameworks, multi-channel EEG signals are tokenized into discrete vector embeddings, allowing large-scale, self-attention-driven architectures (such as bidirectional Encoder Representations from Transformers (BERT) (Nogales et al., 2022) or autoregressive Generative Pre-trained Transformer (GPT) variants) (Kuruppu et al., 2026) to model the contextual “syntax” of brain dynamics. By treating sequences of neural states similarly to semantic phrases, these systems track complex, long-range spatial-temporal distributions across diverse recording environments (Ma et al., 2026).

Current empirically supported applications of LLMs in clinical EEG are primarily restricted to high-level automated report generation and clinical workflow assistance. In these pipelines, pre-extracted numeric EEG metrics or statistical feature vectors are converted into structured text prompts (Li L. et al., 2026), Fine-tuned language models (e.g., domain-adapted Large Language Model Meta AI (LLaMA) or GPT variants) then process these summary prompts to draft standardized clinical EEG reports and preliminary diagnostic summaries, reducing manual documentation time for clinicians while maintaining human-in-the-loop oversight (Levitt et al., 2023).

Emerging studies explore the direct tokenization of multi-channel continuous EEG time-series into discrete vector embeddings. By framing sequences of neural states analogously to semantic tokens, self-attention architectures (such as EEG-BERT or foundational biosignal transformers) learn cross-modal representations and contextual dependencies across recording environments (Chandrasekharan and Jacob, 2025). These approaches show promise in zero-shot or few-shot cross-subject classification and self-supervised representation learning.

The deployment of generative AI and LLMs for direct, autonomous raw signal denoising, automated disease diagnosis, or unassisted clinical decision support remains a future perspective. These applications currently lack sufficient empirical validation from rigorous, multi-center EEG-specific studies. Also, generating raw EEG data directly is risky because AI can create fake brainwaves or incorrect text summaries that look real. Therefore, these models must be thoroughly tested before being used in hospitals (Babu et al., 2025).

### 3.7 State-of-the-art benchmarking and performance synthesis

To provide a clear, comprehensive picture of how different deep learning architectures perform across various clinical domains, it is necessary to benchmark existing literature’s empirical findings. Table 3 systematically compiles and reviews recent state-of-the-art developments, organizing them by model family, targeted brain disorder, used dataset, and specific evaluation metrics.

**TABLE 3:** State-of-the-art comparative synthesis of deep learning architectures applied to clinical EEG neurological disease diagnosis.

References	Brain disorder	Methodology	Dataset	Experimental results
Li et al., 2025	Neurodegenerative diseases	NeuroPredictNet,Adaptive Knowledge Integration Strategy (AKIS)	Sleep-EDF, AMIGOS, PhysioNet Motor Imagery (MI), and MODA benchmark datasets	Achieved up to 90.93% accuracy (Sleep-EDF), 97.90% F1-score (AMIGOS), 97.69% accuracy (PhysioNet MI), and 81.23% accuracy (MODA)
Cansiz et al., 2025	Alzheimer’s Disease (AD)	Deep Ensemble Learning (DEL) model combining 5 different 2D-CNN classifier	Two public EEG datasets for Alzheimer’s Disease (AD) and Healthy Controls (HC) provided by Dr. Dennis Duke from Florida State University	Average classification accuracy: ~97.9%
He et al., 2024	Multiple brain conditions	Unified Convolutional Sparse Transformer (UCST)	Multiple EEG tasks from varied sources	High accuracy
Li K. et al., 2024	Parkinson’s disease (PD) vs healthy controls (HC)	Deep CNN-LSTM	EEG recordings from PD patients and demographically matched healthy controls	Parallel model: Accuracy 98.6 %, Sensitivity 97.1 %, Specificity 97.6 %; Series model: Accuracy 99.7 %, Sensitivity 98.5 %, Specificity 99.1 %
Chen et al., 2023	Alzheimer’s disease (AD), Frontotemporal dementia (FTD)	Combining CNN + Vision Transformer (ViT)	Resting-state EEG data from the OpenNeuro database	80.23 % classification accuracy in distinguishing AD, FTD, and NC subjects
Xu et al., 2022	Epilepsy — preictal seizure prediction	Generative Adversarial Networks (GAN)	Bonn University EEG dataset & CHB-MIT scalp EEG dataset	accuracy improved from ~73.0% to ~78.0% and AUC from 0.676 to 0.704
Choi et al., 2021	Stroke disease	Data preprocessing included raw values, power values (via FFT) and relative values; then they trained deep-learning time-series models (LSTM, Bidirectional LSTM, CNN-LSTM, CNN-Bidirectional LSTM)	Real time data from Six EEG channels: Fz, T7, C1, C2, T8, Oz. Sampling rate 1000 Hz.	Best performance ~ 89.2% (Bidirectional LSTM) for accuracy
Oh et al., 2020	Parkinson’s Disease (PD)	13-layer Convolutional Neural Network (CNN)	EEG recordings from 20 Parkinson’s patients and 20 healthy (normal) subjects	Accuracy of 88.25%, sensitivity of 84.71%, and specificity of 91.77%
Abir et al., 2025	Major Depressive Disorder, Bipolar Disorder, Schizophrenia	EEG preprocessing (ICA, segmentation), ML & DL comparison, hybrid CNN–RNN framework	Public EEG datasets of psychiatric patients	Hybrid CNN–RNN achieved 92% accuracy, 91% sensitivity, 90% specificity, AUC = 0.92
Kashefi Amiri et al., 2025	Epilepsy / Epileptic seizures	Discrete Wavelet Transform (DWT) + 1D Convolutional Neural Network (CNN) + Long Short-Term Memory (LSTM)	TUSZ (Temple University Hospital Seizure Corpus), BONN dataset, CHB-MIT dataset	Accuracy: 94.32% (TUSZ), 97.24% (BONN), 96.94% (CHB-MIT)
Abir et al., 2025	Psychiatric disorders (including MDD)	Random Forest and SVM,, hybrid CNN–RNN framework	Publicly available EEG datasets related to psychiatric conditions	Best Model Performance : CNN(>92%)Sensitivity~91% Specificity~90%
Sarwer et al., 2025	Alzheimer’s disease and schizophrenia.	Deep learning (CNN & LSTM) models	EEG data from patients with Alzheimer’s disease and schizophrenia	CNN/LSTM ~94% accuracy for Alzheimer’s and ~91% accuracy for schizophrenia
Saimon et al., 2025	Parkinson’s disease, epilepsy, and multiple sclerosis (MS).	Multiple machine learning models (SVM, KNN, Gradient Boosting, Random Forest) wavelet feature extraction, PCA, model training, hybrid SVM-KNN	Public EEG and clinical datasets	89–91% accuracy, 90% sensitivity, 88% specificity, and high AUC (~0.95). Combined EEG + clinical data improved accuracy to ~91%.
Nayana et al., 2025	Frontotemporal dementia (FTD), mild cognitive impairment (MCI), and AD	Randomorest (ML), 1D-CNN, 2D-CNN (deep learning)	EEG datasets of participants with AD, FTD, MCI, and healthy controls	2D-CNN achieved 91.13% accuracy outperforming RF (84.78%)
Khan et al., 2025	Alzheimer’s Disease (AD), Frontotemporal Dementia (FTD)	Temporal Convolutional Network (TCN) + Long Short-Term Memory (LSTM SHAP (SHapley Additive exPlanations	EEG recordings from 88 subjects: 36 AD, 29 healthy, 23 FTD - 19-channel EEG, eyes-closed resting state, filtered 0.5–60 Hz, sampled at 500 Hz	Parallel CNN–LSTM: Accuracy = 98.6 %, Sensitivity = 97.1 %, Specificity = 97.6 % - Series CNN–LSTM: Accuracy = 99.7 %
Bansal et al., 2025	ADHD (Attention-Deficit/Hyperactivity Disorder)	Autoencoder + Reptile Search Algorithm (RSA) + Modified ResNet with Double Augmented Attention	EEG data from 121 children (7–12 years): 61 ADHD, 60 healthy controls	Accuracy: 99.42%, Precision: 99.03%, Recall: 99.82%, F1-score: 99.42%, AUC ≈ 0.99
Şentürk, 2025	Chronic neuropathic pain (CNP) etiologies	Deep autoencoders for feature extraction + hybrid Mamba classifier	EEG dataset from 36 patients balanced with SMOTE algorithm	Achieved ~99 % accuracy, AUC = 0.99 across all CNP categories, outperforming SVM (AUC 0.97) and logistic regression (AUC 0.83)

The table summarizes model topologies, target pathologies, datasets, evaluation levels, validation protocols, and standardized performance metrics. Architectures and methods: 1D-CNN/2D-CNN, One/Two-Dimensional Convolutional Neural Network; LSTM, Long Short-Term Memory Network; BiLSTM, Bidirectional LSTM; TCN, Temporal Convolutional Network; ViT, Vision Transformer; UCST, Unified Convolutional Sparse Transformer; GAN, Generative Adversarial Network; SVM, Support Vector Machine; RF, Random Forest; KNN, $k$-Nearest Neighbors; DWT, Discrete Wavelet Transform; FFT, Fast Fourier Transform; ICA, Independent Component Analysis; PCA, Principal Component Analysis; SMOTE, Synthetic Minority Over-sampling Technique; SHAP, SHapley Additive exPlanations; RSA, Reptile Search Algorithm. Pathologies and cohorts: AD, Alzheimer’s Disease; FTD, Frontotemporal Dementia; MCI, Mild Cognitive Impairment; PD, Parkinson’s Disease; MDD, Major Depressive Disorder; ADHD, Attention-Deficit/Hyperactivity Disorder; CNP, Chronic Neuropathic Pain; MS, Multiple Sclerosis; HC/NC, Healthy Controls/Normal Controls. Datasets and metrics: TUSZ, Temple University Hospital Seizure Corpus; CHB-MIT, Boston Children’s Hospital–MIT Scalp EEG Database; MODA, Massive Online Data Repository; AUC/ROC-AUC, Area Under the Receiver Operating Characteristic Curve; F1-Score: Harmonic mean of Precision and Sensitivity.

## 4 Discussion

A critical evaluation of the literature reveals that the perceived dominance of deep learning over conventional approaches is highly context-dependent. A quantitative synthesis shows that while deep architectures (CNNs, Transformers, and hybrid models) achieve higher peak performance metrics (F1-scores > 0.92) on massive, multi-subject datasets, traditional ML algorithms maintain a strong presence in clinical neuroinformatics (Khandekar, n.d.). For instance, classical SVM classifiers paired with non-linear entropy and wavelet features achieve 86–90% sensitivity in seizure and encephalopathy detection while requiring a fraction of the computational footprint (Kalogeropoulos et al., 2026). Furthermore, traditional ML pipelines are naturally resilient to small sample sizes and offer direct feature transparency, whereas CNNs and deep networks require explicit *post-hoc* explainability tools (e.g., SHAP, LIME, or attention maps) to justify their predictions to clinical practitioners.

The application of deep learning to electroencephalography (EEG) analysis has significantly advanced automated neurodiagnostics. However, translating these architectures from controlled laboratory environments to clinical practice requires an understanding of their performance claims, methodological vulnerabilities, and the clear distinction between limitations intrinsic to specific model families versus those originating from implementation and dataset constraints. To guide future research, it is vital to separate the limitations that are structurally intrinsic to an architectural design from those that arise dynamically due to implementation and data choices.

Convolutional neural networks (CNNs) excel at extracting features from small, localized windows of data, making them highly effective at spotting sudden changes like sharp epileptic spikes on an EEG (Jemal et al., 2022). However, this hyper-focused design means they struggle to model long-range temporal context, which is an intrinsic limitation that causes them to fail at connecting events over long periods or mapping relationships between distant areas of the scalp (Liu et al., 2023). In contrast, their tendency to generate overly smoothed data that blurs vital, tiny brain waves such as high-frequency gamma bands is an implementation choice driven by the common use of standard Mean Squared Error (MSE) loss functions. Furthermore, their inability to adapt to changing electrode configurations reflects insufficient training data diversity rather than an uncorrectable architectural flaw (Luo et al., 2020).

Recurrent networks (like RNNs, LSTMs, and GRUs) (Rivas et al., 2025) process EEG data sequentially one sample at a time, their design creates a mathematical bottleneck that prevents GPUs (Raimondo et al., 2012) from processing data in parallel, leading to significantly long training cycles. This step-by-step chain reaction means that a single high-amplitude movement artifact can completely corrupt the network’s internal memory, causing errors to cascade across all subsequent samples. Beyond these structural flaws, these models suffer from practical, implementation-driven limits; for instance, longer data windows often cause gradients to vanish, meaning the network effectively stops learning. Furthermore, because these networks rely on a large number of internal parameters, training them on small datasets creates a high risk of overfitting, where the model simply learns to replicate the training data instead of learning true brain patterns (Algarni et al., 2022).

Autoencoders take a different approach by using an architectural information bottleneck to compress data and filter out noise, but this design comes with an intrinsic constraint: the compression is inherently lossy. It forces complex EEG data into abstract, hidden representations that are incredibly difficult for researchers to map back to real biological or brain mechanisms (Vincent et al., 2008). On the practical side, autoencoders are completely dependent on the specific data distributions they are trained on. For example, a model optimized purely on uniform, resting-state data will severely distort signals when applied to complex sleep or cognitive data, because it forces those entirely new brain dynamics into its rigid, over-constrained bottleneck (Wang et al., 2026).

Generative adversarial networks (GANs) (Tibermacine et al., 2025) clean signals and augment data using a competitive game. Structurally, their main built-in flaws are high training instability and a vulnerability to catastrophic mode collapse, a failure where the model becomes trapped generating the same waveform pattern repeatedly instead of showing the true variety of brain signals. In contrast, the major risk of the model hallucinating false brain waves or erasing genuine pathological biomarkers is a setup flaw. This issue is caused by poor hyperparameter tuning or a lack of strict loss rules during training, rather than an unfixable problem with the framework itself.

Transformer-based models drop step-by-step loops entirely and use self-attention to track relationships across time and channels, making them highly effective at spotting irregular noise bursts across the scalp. However, this design comes with clear intrinsic trade-offs: their memory demand grows quadratically with signal length, making long, high-density EEG data computationally expensive, and they lack built-in rules for naturally understanding continuous time sequences. Additionally, their strong tendency to overfit is a reflection of small, scarce EEG datasets rather than a structural flaw. While their complex parameter structures limit immediate transparency, this can be managed by integrating native attention maps to trace the model’s focus directly back to physical electrode regions on the brain (De la Rosa et al., 2026).

A major problem in this field is that the high-performance metrics reported in labs are often artificial illusions caused by data leakage and poor testing methods, which give highly misleading results when clinical datasets face severe class imbalances. To achieve true clinical utility and combat these dataset imbalances, the field must stop looking at accuracy alone and consistently report sensitivity, specificity, balanced accuracy, and F1-scores across different evaluation levels, including window-, segment-, subject-, and event-level partitions. When continuous EEG records are split randomly by time segments instead of keeping patient data entirely separate, the model simply memorizes individual biometric signatures or background noise patterns instead of learning the actual signs of a disease (Brookshire et al., 2024). This causes a drastic drop in accuracy when the model is tested on new subjects, different age groups, or new hospital equipment, a failure that is not a limitation of the models themselves, but a direct flaw in the validation strategy chosen by researchers. Toward this end, the field must transition to rigid, subject-independent testing and utilize much larger, diverse open-access datasets (Tegenaw et al., 2026). For example, in seizure detection, standard window accuracy is insufficient; medical practitioners need to see event-based metrics like true event sensitivity, false alarms per hour, detection delays, and the exact moments a seizure starts and ends (Acharya et al., 2018; Sudhakar Jebaraj and Elango, 2025). Similarly, for neurodegenerative conditions like Alzheimer’s or Parkinson’s disease, models must be tested on their ability to classify whole subjects and handle massive differences between individual baseline brain waves. For sleep disorders, systems need to accurately track sudden transitions between sleep stages epoch-by-epoch rather than just taking a generic average. Finally, the field must strictly separate personalized, patient-specific training which uses a patient’s own past data to predict their future trends from rigid subject-independent testing. True clinical robustness can only be proven using cross-dataset testing and Leave-One-Subject-Out (LOSO) validation to ensure models are identifying genuine clinical indicators instead of just memorizing a specific patient’s biometric signature.

Crucially, the field must stop mixing up the goals of artifacts, reconstructing signals, extracting features, and classifying diseases, as each task has completely different purposes and evaluation rules. Denoising and reconstruction networks are built to clean data and must be measured using signal-quality metrics like Mean Squared Error (MSE), Signal-to-Noise Ratio (SNR) (Sudhakar Jebaraj and Elango, 2025) improvements, and waveform correlation coefficients. On the other hand, diagnostic models are designed to spot diseases and require classification or event-detection metrics like sensitivity, specificity, and F1-scores. Researchers often assume that a cleaner, visually perfect EEG signal automatically means an AI will make a better medical diagnosis. However, this is not always true; aggressive cleaning filters can easily erase tiny, chaotic brain waves that look like noise to a reconstruction network but actually contain the exact hidden keys needed for an accurate diagnosis. Therefore, future studies must evaluate these steps separately to prove that cleaning choices actually help, rather than accidentally ruin, downstream diagnostic accuracy (Tawhid et al., 2023).

Moving these advanced methods into real hospital settings also requires overcoming severe operational barriers regarding interpretability, domain shifts, and deployment hardware. The “black box” complexity of deep learning severely harms clinical trust because medical practitioners cannot easily verify how a model reaches a decision (Moutonnet et al., 2024). However, this interpretability barrier is an addressable design limitation; integrating post-processing tools such as SHAP values or saliency maps allows model decisions to be traced back to identifiable brain regions. Furthermore, real clinics feature major domain shifts, such as changing electrode-skin impedance, different hardware sampling rates, and environmental noise. Poor performance under these shifts is an implementation failure caused by a lack of robust preprocessing or domain adaptation during training. Finally, the massive computing demands of transformers restrict their use in real-time clinical deployment, requiring engineering fixes like model compression or quantization to fit onto lightweight bedside monitors.

Emerging large language model (LLM) approaches and multimodal systems represent a promising frontier, but their clinical readiness must be critically evaluated by separating demonstrated findings from speculative potential (Chandrasekharan and Jacob, 2025). While automated clinical report generation from pre-calculated EEG summaries is empirically demonstrated, direct applications to autonomous diagnosis, denoising, and unassisted clinical decision support remain prospective and lack rigorous empirical backing in EEG-specific cohorts. Foundation models attempting zero-shot translation on raw time-series data face severe risks of generative hallucinations, wherein synthetic wave features or clinical metrics lack physical ground truth (Babu et al., 2025). Overcoming this barrier requires transitioning from conceptual frameworks to rigorous empirical validation across diverse clinical datasets, incorporating multi-model verification protocols and constant human expert oversight to ensure absolute patient safety (Yu et al., 2025).

## 5 Conclusion

This review highlights the rapid evolution of EEG analysis driven by deep learning, generative, and attention-based models. Across multiple neurological and psychiatric disorders, CNN, RNN, GAN, and transformer-based approaches consistently outperform traditional machine learning methods, achieving high accuracy and automatic feature learning with minimal reliance on manual feature engineering. Hybrid configurations and end-to-end architectures have significantly advanced the capacity to handle non-stationary, artifact-contaminated neural time-series data while preserving underlying cerebral dynamics. Emerging integrations with attention mechanisms and LLM-inspired frameworks further indicate a transformative shift toward automated, scalable, and context-aware clinical interpretation.

Despite these computational advancements, major methodological and translational gaps continue to restrict real-world clinical adoption. The field is severely bounded by a persistent lack of standardized evaluation, a scarcity of uncorrupted human ground-truth records, the black-box opacity of deep networks, data leakage vulnerabilities during subject-dependent testing, and the severe diagnostic risk of generative hallucinations. Future research trajectories must move away from simply describing high laboratory performance metrics on closed datasets. Instead, the neuroinformatics community must prioritize rigorous cross-dataset validation, robust subject-independent benchmarking, explainable AI workflows, and hardware-efficient edge models to guarantee accuracy, reproducibility, and safety in precision clinical diagnostics.
